# Mathematical Modeling of Release Kinetics from Supramolecular Drug Delivery Systems

**DOI:** 10.3390/pharmaceutics11030140

**Published:** 2019-03-21

**Authors:** Constantin Mircioiu, Victor Voicu, Valentina Anuta, Andra Tudose, Christian Celia, Donatella Paolino, Massimo Fresta, Roxana Sandulovici, Ion Mircioiu

**Affiliations:** 1 Department of Applied Mathematics and Biostatistics, Faculty of Pharmacy, “Carol Davila” University of Medicine and Pharmacy, Bucharest 020956, Romania; constantin.mircioiu@yahoo.com (C.M.); andratds@yohoo.com (A.T.); 2 Department of Clinical Pharmacology, Toxicology and Psychopharmacology, Faculty of Medicine, “Carol Davila” University of Medicine and Pharmacy, Bucharest 020021, Romania; victor.voicu@yahoo.com; 3 Department of Physical and Colloidal Chemistry, Faculty of Pharmacy, “Carol Davila” University of Medicine and Pharmacy, Bucharest 020956, Romania; 4 Department of Pharmacy, G. D’Annunzio University of Chieti–Pescara, Chieti 66100, Italy; c.celia@unich.it; 5 Department of Clinical and Experimental Medicine, “Magna Græcia” University of Catanzaro, 88100 Germaneto (CZ), Italy; paolino@uniczt.it; 6 Department of Health Sciences, School of Pharmacy, “Magna Græcia” University of Catanzaro, 88100 Germaneto (CZ), Italy; fresta@uniczt.it; 7 Department of Applied Mathematics and Biostatistics, Titu Maiorescu University, Bucharest 004051, Romania; roxana.sandulovici@yahoo.com; 8 Department of Biopharmacy and Pharmacokinetics, Titu Maiorescu University, Bucharest 004051, Romania; ionutu@yahoo.com

**Keywords:** boundary conditions, diffusion equation, drug carriers, release kinetics

## Abstract

Embedding of active substances in supramolecular systems has as the main goal to ensure the controlled release of the active ingredients. Whatever the final architecture or entrapment mechanism, modeling of release is challenging due to the moving boundary conditions and complex initial conditions. Despite huge diversity of formulations, diffusion phenomena are involved in practically all release processes. The approach in this paper starts, therefore, from mathematical methods for solving the diffusion equation in initial and boundary conditions, which are further connected with phenomenological conditions, simplified and idealized in order to lead to problems which can be analytically solved. Consequently, the release models are classified starting from the geometry of diffusion domain, initial conditions, and conditions on frontiers. Taking into account that practically all solutions of the models use the separation of variables method and integral transformation method, two specific applications of these methods are included. This paper suggests that “good modeling practice” of release kinetics consists essentially of identifying the most appropriate mathematical conditions corresponding to implied physicochemical phenomena. However, in most of the cases, models can be written but analytical solutions for these models cannot be obtained. Consequently, empiric models remain the first choice, and they receive an important place in the review.

## 1. Introduction

Supramolecular drug systems (SMDS) constitute a very wide concept. Supramolecular chemistry can be understood as the “chemistry of multi-molecular complexes”, i.e., approximately whole chemistry. Since such an approach would push the discussion beyond the concrete aspects in the meta-science domain, the present review aims to focus on microscopic, multiphasic drug carriers. 

No matter what their physicochemical and pharmaceutical form might be, drug systems should have two essential characteristics: biocompatibility and controlled release capacity. Controlled release is imposed by the necessity of providing a therapeutic agent at the action site in the therapeutic window, i.e., between efficacy and toxic levels. The site of action is generally unknown, but it is commonly accepted that the active substance is transported to the “receptor” trough the bloodstream. Under those circumstances, the less ambitious but more feasible task of the majority of research and development activities is oriented toward pharmacokinetic goals, by means of pharmaco/toxico kinetics and dynamic modeling.

When the pharmacokinetic windows are not known, a series of technological factors and methods are used for assurance of controlled release kinetics of active substances. 

In vitro evaluation of the results, correlation with in vivo results, and finally the prediction of pharmacokinetics of active substances involve high-level mathematical models and methods developed for describing the so-called “mass transfer phenomena”. 

It is to underline that mathematical models involved in the description of mass transfer are common methods for describing all type of transfer phenomena. The phenomena of mass, heat, and impulse transfer are quite different fields in physics but are described by practically identical mathematical equations. From the point of view of material support of transfer, these phenomena are classified as follows:

- diffusion phenomena, when the support is represented by molecules;
- convective transport by currents in fluids, described by fluid mechanics;
- radiative transport of elementary particles.

Mathematical equations and methods are in fact common for all these processes. In fluid mechanics, we have the Navier–Stokes equation, and, in case of heat transfer, we have the Fourier–Kirchhoff equation. 

As a consequence of the above-presented similarities there are also correspondences between models. For example, almost all solutions of the heat transfer equation and from fluid mechanics are transferrable, and they are indeed applied in the analysis of diffusion phenomena.

As underlined by Crank in “The Mathematics of Diffusion” [1], all heat transfer solutions are translated into mass transfer solutions, replacing the constant k (thermal diffusivity) with another constant, D ( the diffusion coefficient). In the preface of the first edition, the author stated that “the mathematical theory of diffusion is founded on that of heat conduction” and, correspondingly, the early part of this book was developed based on “Conduction of heat in solids” by Carslaw and Jaeger” [2]. In fact, the main ideas come from the theory of heat transfer by Fourier published in 1822 and later applied to diffusion by Fick in 1855.

A fundamental characteristic of transfer phenomena modeling is that the solutions are very “smooth” functions, having a behavior in the domain where transfer takes place, determined by their values on the frontier. It is essential to take note that frontiers in both in vitro and in vivo transfer phenomena are most frequently “the interfaces”. The authors of the present paper considered in a series of research papers that even pharmacodynamic effects are most frequently the results of accumulation and effects at interfaces and particularly “membrane interfaces” [3]. 

All models of transfer phenomena are described by differential equations, and the solutions of real practical interest are particular solutions, defined by properties on frontiers called “boundary conditions”.

The present paper attempts to analyze the release kinetics from supramolecular drug delivery systems, starting from boundary conditions determined by the phenomenological conditions, simplified and idealized in order to lead to problems which can be analytically solved.

## 2. Mathematical Methods for Solving Transfer Equations in Initial and Boundary Conditions Imposed by Particular Systems

### 2.1. Diffusion Equation

The evaluation of in vitro and in vivo release kinetics of active substances from drug systems plays an important role in predicting and management of both efficacy and safety. Kinetics is more than a scientific goal; it is an essential quality parameter of all type of drugs. 

Keeping in mind the high diversity of supramolecular drug systems and apparently huge number of phenomenological local characteristics, a classification of models seems to be an impossible task.

On the other hand, in practically all these release processes, the diffusion phenomenon is involved, described by the diffusion equation.

(1) $$\frac{\partial c}{\partial t}=D\frac{\partial^{2}c}{\partial x^{2}}.$$

In this form, the equation has an infinite number of solutions, including the banal solution c(x,t)=0.

The above equation only makes sense when the problem concerns a solution satisfying some “initial and boundary conditions”. Cauchy’s problem refers to the existence and uniqueness of solutions for given coefficients and boundary conditions. Unfortunately, the number of methods for solving the equation is low, and analytical solutions (i.e., solutions described explicitly by functions) can be obtained only in simple initial and boundary conditions. On the other hand, after some simplifications and idealizations, a great number of different release processes lead to these “good conditions”. Since meaningful solutions are connected with the initial and boundary conditions, the modeling of release kinetics consists of identifying the most appropriate mathematical conditions connected with the implied physicochemical phenomena.

Consequently, a natural classification of the release models (or at least of the quantitative ones) has to start from the geometry of the diffusion domain and its frontier and from initial conditions. Last but not least, frontiers are most frequently boundaries between subdomains, where discontinuities and critical changes of physicochemical properties are the rule. 

### 2.2. Initial and Boundary Conditions

Boundary conditions (BC) were classified more than a century ago in standard papers on the theory of heat transfer, and this classification was translated into the mass transfer case by Crank [1] as follows:

BC1. A surface having a prescribed concentration, in contact with a medium with a concentration proportional with that of surface, is defined by a partition coefficient, similar to the case with equilibrium between a liquid and its vapors.

BC2. A flux across interface $-D\frac{\partial c}{\partial x}=F(t)$ (Neumann condition).

BC3. An impermeable surface $\frac{\partial c}{\partial x}=0$.

BC4. Newton’s law of cooling, or “radiation boundary condition”, a flux proportional to the difference in temperature between surface and medium, which, on other hand, is equal to the heat loss in the direction of normal to the surface. In terms of concentration, the condition is

(2) $$\frac{\partial C}{\partial n}+\alpha \left(C_{s}-C_{0}\right)=0.$$

The transfer across a membrane of thickness *l* corresponds to $\frac{\partial C}{\partial x}+\alpha \left(C_{s}-C_{0}\right)=0$ for *x* = *l* and $-\frac{\partial C}{\partial x}+\alpha \left(C_{s}-C_{0}\right)=0$ for *x* = 0.

BC5. Transfer from a well-stirred release medium of fixed volume *V* and uniform concentration leads to the following condition:

(3) $$V\frac{\partial c}{\partial t}=D\frac{\partial c}{\partial x}\;x=0.$$

BC6. The conservation principle at an interface between two media of different properties gives

(4) $$D_{1}\frac{\partial C_{1}}{\partial x}=D_{2}\frac{\partial C_{2}}{\partial x}\;C_{1}=PC_{2}+Q,$$

where *P* and *Q* are two constants.

BC7. If in the medium exists as a source of substance (for example, a chemical reaction) at a rate per unit volume *A*, the boundary condition is

(5) $$\frac{\partial c}{\partial t}=D\frac{\partial^{2}c}{\partial x^{2}}+A.$$

BC8. Moving boundary conditions could be considered as issuing from the immobilization of molecules in pores or holes. Boundaries changing in time, *X*(*t*), are connected to the flux coming to or leaving it via the following relationship:

(6) $$-D_{1}\frac{\partial C_{1}}{\partial x}+D_{2}\frac{\partial C_{2}}{\partial x}=L\frac{dX}{dt},$$

where *L* is the capacity of the immobilizing site in the unit volume for diffusing molecules. 

Diffusion phenomena are essentially implicated in all release kinetics from practically all pharmaceutical formulations. Solutions of diffusion equations have, in this context, a great importance for all models describing and predicting the evolution of drug concentration in release media. In the non-mathematical literature, it is customary to present direct solutions associated with different phenomenological conditions without specification of initial and boundary conditions. 

A classification of models as a function of initial and boundary conditions is useful for at least two reasons, shown below.

1. Different combinations of phenomenological conditions can lead to the same initial and boundary conditions and, consequently, to the same mathematical solutions. It frequently happens that experimentally determined release kinetics to fit a theoretical law are deduced in completely different phenomenological conditions.
2. Derivation of solutions essentially implies the initial and boundary conditions, such that the in-depth analysis of phenomena and prediction possibilities are best achieved in connection with understanding of the mathematical aspects.

As discussed in the section below, the classification of models starts from initial and boundary conditions for “abstract mathematical models”, and evolves toward evaluation of abstract models in phenomenological conditions compatible with the mathematical conditions. In all cases, the extent of similarity between real and ideal mathematical conditions with time course changes of phenomenological conditions is examined. 

Boundaries in drug systems are usually interfaces; however, for mathematical reasons, it is useful to consider also frontiers “at infinite distance”, in which case the calculus is simplified. Interfaces are fixed or moving boundaries. A particular characteristic of nanosystems is the fact that interfaces are very large and usually curved surfaces, which requires taking into account local domains with discontinuous conditions, leading to “generalized functions” or “distributions” as solutions.

### 2.3. Release in an Infinite Medium from an Interface where Concentration Is Kept Constant: Laplace Transform Method

**Figure 1:**
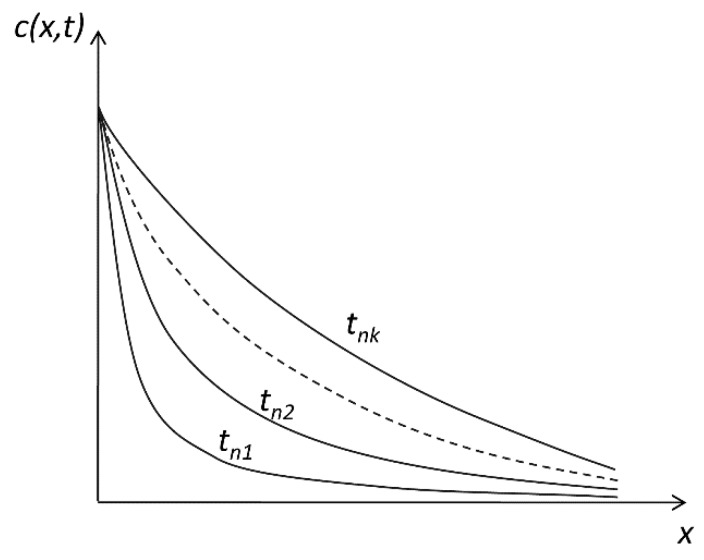
Spatial distribution of the active substance at different (*t_n1_, …, t_nk_*) time points.

Let us use the abbreviation c*_s_* for the constant concentration at the interface. This suggests the case of “saturation concentration” (Figure 1), which helps us concretize mathematical phenomenon; however, the mechanisms for keeping a relative constant concentration at an interface are surely diverse and multiple.

In this case, we have to solve the diffusion equation in the following initial and boundary conditions: $x=0,\;\mathrm{and}\;c\left(0,t\right)=C_{s}$. 

We further consider that the concentration in the release medium is initially zero, i.e., t=0 and c(x,0)=0.

Since the diffusion front advances with a finite velocity, in whatever time *t*, if we go far enough from the interface, the concentration will be zero, which, in mathematical terms, can be written as

(7) $$x=\infty \;\underset{x\to \infty }{\lim}c(x,t)=0.$$

In these conditions, we obtain that the flux of drug across the interface is proportional to the square root of time. We can further compute the quantity *Q*(*t*) of drug transferred after time *t* across interface *x* = 0.

(8) $$J=\frac{1}{A}\frac{dm}{dt}\;\mathrm{and}\;\int_{0}^{t}Jdt=\int_{0}^{t}\frac{1}{A}\frac{dm}{dt}dt=\frac{Q(t)}{A};$$

(9) $$\frac{Q(t)}{A}=\int_{0}^{t}Dc_{s}\frac{1}{\sqrt{\pi Dt}}dt=\frac{c_{s}}{\sqrt{\pi }}\sqrt{D}2\sqrt{t}=c_{s}\frac{2}{\sqrt{\pi }}\sqrt{Dt}.$$

As a simple experimental model to verify these laws, we can consider the dissolution of an active substance or a drug formulation placed at the bottom of a vessel. If the concentration in the formulation is much higher than the solubility of the active substance, the concentration at the interface with the dissolution medium will actually be the saturation concentration $c_{s}$. If the height of a vessel is enough to assure that, in the time interval in which we are interested, the front of the substance does not reach the upper surface, we comply with the conditions of the above mathematical model. 

Experimentally, this law leads to a linear dependence of the released amount of active substance, proportional to the square root of time. Such a dependence of the released amount on the square root of time is frequently obtained in literature; generally, the authors consider that this is the case of the Higuchi square root law, although phenomenological conditions of the respective experiment are very different from those used by Higuchi. 

Bolisetti et al. [4] empirically tested a number of models fitting the release data of repaglidine from floating gels of cubosomes and concluded that release follows Higuchi’s law. In the release studies of coumarines from nanostructure-loaded mesoporous silica, Al-Kady et al. also presumed a Higuchi model [5]. Many other examples of such dependence are presented in the second part of this paper.

### 2.4. Transfer at Liquid/Liquid Interfaces: Release from Microemulsions

#### 2.4.1. Stationary State Models

In recent years, emulsions and self-emulsifying drug delivery systems were increasingly used to enhance the oral bioavailability of poorly water-soluble drugs, especially of highly lipophilic ones [6,7,8,9,10,11,12]. The release from micro- and nanoemulsions can be considered as a direct application of transfer across liquid/liquid interfaces.

In fact, in the case of microemulsion, there is not a simple oil/water interface since the formation of stable emulsions is not possible without surface active agents which accumulate at the interface forming a monolayer. The study of the stability of microemulsions has to include both thermodynamic and kinetics aspects [13]. For measuring the release of active substances from microemulsions, two experimental methods are generally used: the membrane diffusion technique and the in situ method [14].

**Figure 2:**
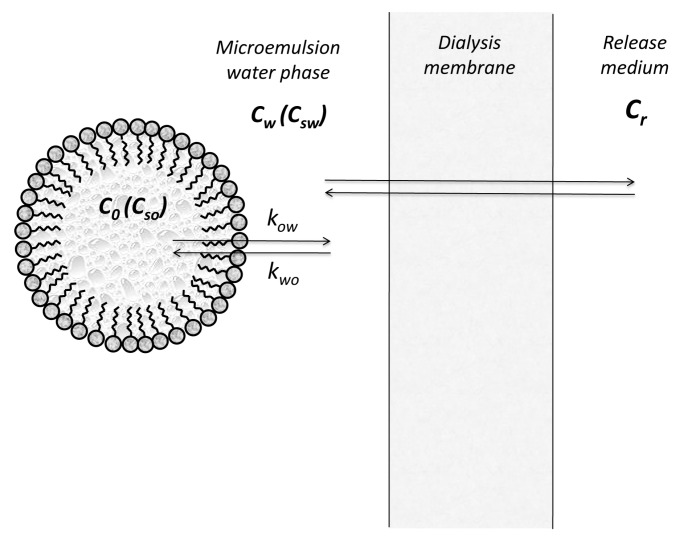
Release kinetics of a drug from microemulsions in an experiment using a dialysis membrane.

In the membrane diffusion models, we consider the drug partition between oil droplets, micelles, and the aqueous continuous phase, as well as transfer across the membrane separating the microemulsion from the release medium. By retaining from all involved processes only interfacial transfers from oil and water (Figure 2), Yotsuyanagy and Higuchi [14,15] expressed the fluxes as $\Phi_{ow}=k_{ow}C_{o}$ and $\Phi_{wo}=k_{wo}C_{w}$, where $C_{w}$ and $C_{o}$ represents the concentrations of drug in water and oil phases, respectively.

Friedman and Benita [16] evaluated the release of morphine from emulsions, considering distribution in three phases: the continuous aqueous phase, the oil droplets, and the surfactant micelles.

The fluxes decrease to zero when $C_{w}$ approaches saturation value ($C_{s}$), and the concentration in oil is zero, such that the following equations are satisfied:

(10) $$\Phi_{ow}=k_{ow}\frac{C_{o}}{C_{sw}}\left(C_{sw}-C_{w}\right)\;\mathrm{and}\;\Phi_{wo}=k_{wo}\frac{C_{wo}}{C_{so}}\left(C_{so}-C_{o}\right),$$

where $C_{sw}$ and $C_{so}$ represent the saturation concentrations of drug in water and oil phases, respectively.

In all these models, it is accepted that we can speak about the concentrations in oil and water phases, i.e., the concentrations are uniform all the time.

Applying the same simplifications as above, the transfer across the membrane separating the microemulsion from the release medium, the following formula was proposed for the flux across membrane:

(11) $$\Phi_{p}=k_{ow}\frac{D}{\delta_{m}}\left(k_{pd}C_{w}-k_{pr}C_{r}\right),$$

where $\delta_{m}$ is the membrane thickness, D is the drug diffusion coefficient inside the membrane, *k_pd_* is the drug partition coefficient between membrane and microemulsion aqueous phase, and *k_pr_* is the membrane release medium partition coefficient.

#### 2.4.2. Compartmental Models

Applying the above expressions of fluxes, the differential equations describing the time course of concentrations of drug in water and oil phases are as follows:

(12) $$\frac{dC_{w}}{dt}=A\frac{\Phi_{ow}}{V_{w}}-A\frac{\Phi_{wo}}{V_{w}}-S\frac{\Phi_{p}}{V_{w}};$$

(13) $$\frac{dC_{o}}{dt}=A\frac{\Phi_{wo}}{V_{o}}-A\frac{\Phi_{ow}}{V_{o}}.$$

Grassi et al. [17] applied the above model in the analysis of nimesulide release from microemulsions. The release of nimesulide from microemulsions was also evaluated by Siroti et al. [18], but a more complex model was considered, taking additionally into account the drug transfer from micelles.

The identification of transfer parameters starting from experimental data is an extremely difficult mathematical task, with instability of solutions being the rule rather than the exception.

A simplified approach, more empirical but leading to an easier way to mathematically solve the problem, is to discard the physicochemical significance of coefficients and to retain only the property of linear transfer between three compartments: oil, water, and external medium.

(14) $$\frac{dC_{w}}{dt}=k_{ow}C_{o}-k_{wo}C_{w}-k_{wr}c_{w};$$

(15) $$\frac{dC_{o}}{dt}=-k_{ow}C_{o}+k_{wo}C_{w}.$$

The system can be easily solved by the method of Laplace transform, whereby the solutions are expressed as sums of exponentials.

The model has the advantage that it can be coupled with pharmacokinetic compartmental models for predicting release in vivo and the absorption of active substances. For example, recently, Grassi et al. [19] reviewed such extended models and developed a model for the release of drugs and their percutaneous absorption. 

Finally, at transfer across interfaces, the main resistance could be in the transfer at the interface, in which case the concentration follows a sum of exponential behavior. Mircioiu et al. [20] modeled the transfer of chemical warfare agents and pesticides, such as chlorpyrifos, dichlorvos, or malathion, across the skin and synthetic membranes as first-order kinetic and/or square-root law transfer processes. Results suggested the possibility to apply synthetic membranes for predicting the percutaneous absorption of organophosphorus compounds.

For the in vivo experiments, pharmacokinetics are well enough described by empiric compartmental models [21]; however, in some instances, physiological models similar to in vitro mechanistic models are unavoidable [22].

In the case of the release of anti-tuberculosis drugs from Tween-embedded microemulsions [23], evaluation indicated that the release of pyrazinamide and isoniazid is non-Fickian, whereas it was found to be Fickian for rifampicin. Finally, in deciding the model, both mathematical and phenomenological criteria had to be used simultaneously [24].

### 2.5. Diffusion in Membranes: Method of Separation of Variables

#### 2.5.1. Diffusion in a Domain Bordered by Two Interfaces where Concentration Is Kept Constant

**Figure 3:**
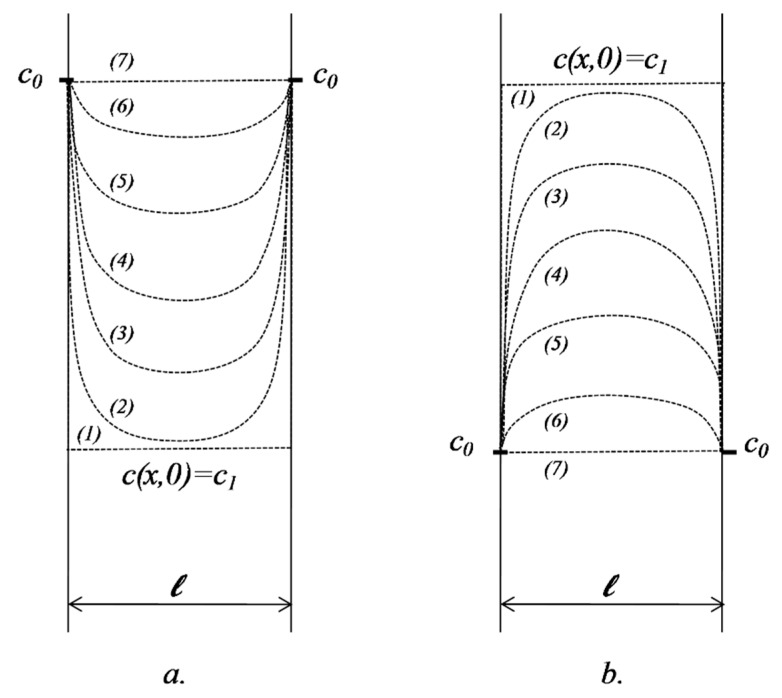
Spatial distribution of the concentrations of active substance at different time intervals: (**a**) case $c_{1}<c_{0}$, transfer into the membrane; (**b**) case $c_{1}>c_{0}$, transfer out of the membrane.

We consider the release from (or into) a domain of thickness 2ℓ, having an initial concentration $c_{1}$ in an environment where the concentration remains constant over time, $c_{0}$ (Figure 3).

If concentration at the point *x* in the matrix at the moment *t* is *c*(*x*,*t*), the initial and boundary conditions can be written in the form below.

(16) $$\left|\begin{array}{c} c_{0}\\ c_{1}\\ c_{0} \end{array}\right|\;\begin{array}{cc} x=2\ell , & c(2\ell ,t)=c_{0}\\ t=0 & c(x,0)=c_{1}\\ x=0 & c(0,t_{0})=c_{0} \end{array}.$$

The diffusion equation to be solved is again Fick’s second law.

The solution of the equation in the specified initial and boundary conditions is

(17) $$\frac{c-c_{0}}{c_{1}-c_{0}}=\frac{4}{\pi }\sum \frac{1}{2k+1}\sin\frac{\left(2k+1\right)\pi x}{2\ell }\;e^{-\frac{{\left(2k+1\right)}^{2}\pi^{2}t}{4\ell^{2}}}.$$

A detailed demonstration of the above expression is presented in Appendix A.

A very important case is when $c_{0}=0$, named in the pharmaceutical literature as “sink” conditions. This situation refers to experiments when, due to the huge volume of the fluid in which the release occurs, the concentration is practically null. We can consider as an example the release of locally applied drugs, e.g., a transdermal therapeutic system, where the bloodstream permanently removes the active substance from the release site. Note that, in biopharmacy, as a general rule, all release/dissolution experiments are planned to be performed in conditions as close to “sink” as possible.

#### 2.5.2. Diffusion in a Domain Bordered by Two Interfaces of Constant but Different Concentrations

**Figure 4:**
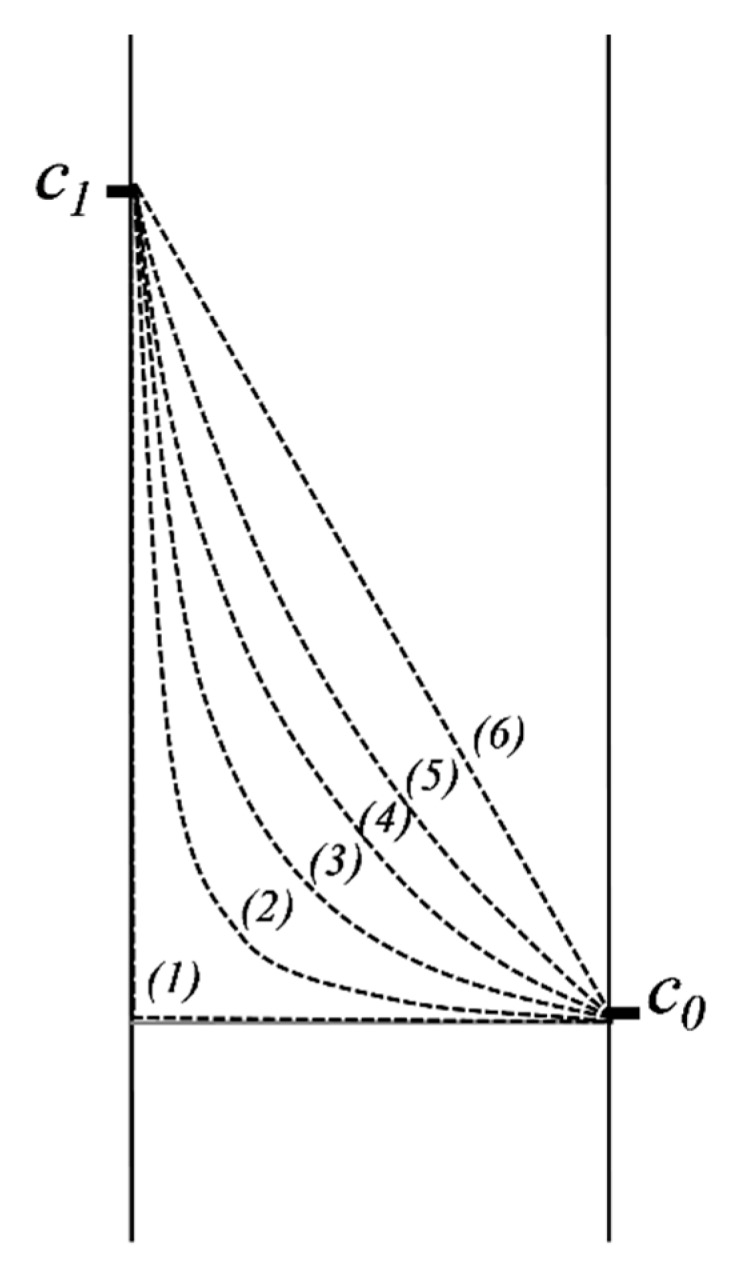
Distribution of concentration in a membrane separating two domains with constant concentrations.

We further consider the case of transfer through a submerged membrane in a fluid medium in which the diffusion process has a much faster rate than the velocity of diffusion in the membrane. If, in addition, we consider that the volume of fluid is very high, we can again approximate that the concentration of active substance remains constant during the experiment (Figure 4). 

Applying again the method of separation of variables, similar to what is presented in Appendix A, the concentration in the membrane is defined by the following formula:

(18) $$\frac{c-c_{0}}{c_{1}-c_{0}}=\frac{x}{\ell }+\frac{-2}{\pi }\sum \frac{{\left(-1\right)}^{n}}{n}\sin\frac{n\pi x}{\ell }e^{-D\frac{n^{2}\pi^{2}t}{\ell^{2}}}.$$

### 2.6. Diffusion Equation in Spherical and Cylindrical Coordinates

#### 2.6.1. Solutions of Diffusion Equation in Spherical Coordinates

**Figure 5:**
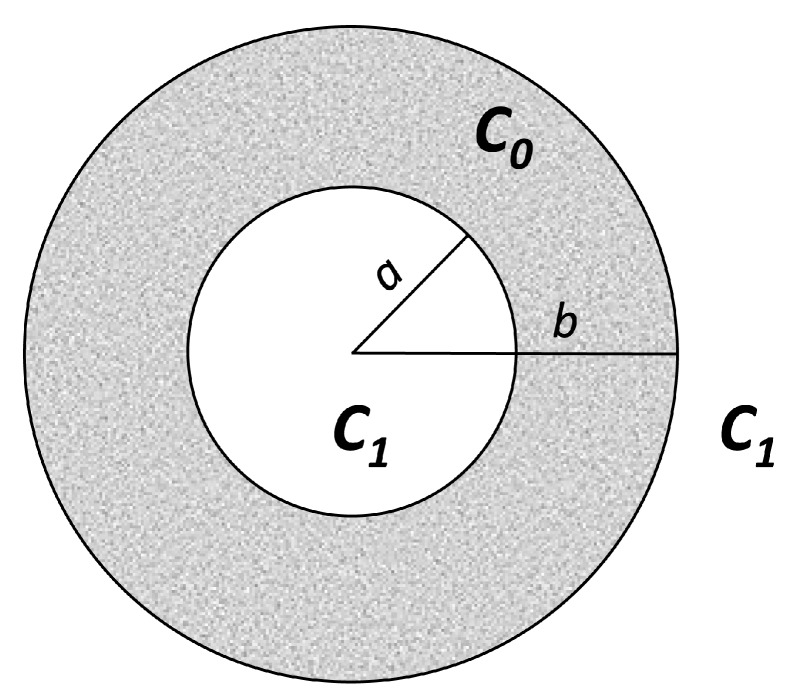
Radial transfer across a hollow sphere in a release medium where the concentration of drug has a constant value $c_{1}$.

In cases when the curvature of interfaces is great it is more appropriate to use spherical coordinates (Figure 5). After writing the Laplacian in spherical coordinates and considering only radial flow, the diffusion equation becomes

(19) $$\frac{\partial C}{\partial t}=D\left(\frac{\partial^{2}C}{\partial r^{2}}+\frac{2}{r}\frac{\partial C}{\partial r}\right).$$

The solution obtained in this case for *c*(*x*,*t*) inside the hollow sphere will be

(20) $$\frac{c-c_{0}}{c_{1}-c_{0}}=1+\frac{2}{\pi r}\sum \left(\frac{b\cos n\pi -a}{n}\right)\sin\frac{n\pi (r-a)}{b-a}\;e^{-\frac{Dn^{2}\pi^{2}t}{{\left(b-a\right)}^{2}}},$$

as previously described by Crank [1] and by Carslaw and Jaeger [2]. The percentage amount of active substance entering (leaving) via the interfaces as a function of time will be

(21) $$\frac{M_{t}}{M_{\infty }}=1-\frac{6}{\pi^{2}\left(a^{2}+ab+b^{2}\right)}\sum {\left(\frac{b\cos n\pi -a}{n}\right)}^{2}e^{-\frac{Dn^{2}\pi^{2}}{{\left(b-a\right)}^{2}}t}.$$

#### 2.6.2. Release from a Non-Degrading Polymer

A materialization of the above model is the release from a “core”, where the concentration of active substance is above the saturation concentration $C_{s}$, and where diffusion occurs across a diffusion membrane in a well-stirred medium.

The release rate of an anti-Parkinson drug from a non-degrading polymer (poly(lactic-co-glycolic acid)—PLGA) was studied [25]. During the time of the experiment, the changes in the volume of the polymer matrix were negligible; therefore, the polymer microspheres were considered as non-biodegradable implants. The released amount followed an equation very similar to the above one, as shown below.

(22) $$\frac{M_{t}}{M_{\infty }}=1-\frac{6}{\pi^{2}}\sum {\left(\frac{1}{n}\right)}^{2}e^{-\frac{Dn^{2}\pi^{2}}{R^{2}}t},$$

which corresponds to the case of *a* = 0 and *b* = *R*.

#### 2.6.3. Release from Lipid Dosage Forms

The release of sodium salicylate from spherical beads based on Gelucire 46/07 (melting point = 46 °C, hydrophilic lipophilic balance value (HLB value) = 7) in simulated gastric fluid was well fitted [26] by the same equation written above.

This is to underline that mathematical conditions are translations of phenomenological initial and boundary conditions associated with the carrier, drug, and release conditions. For example, Siepmann [27] considered, in the case of release from lipid dosage forms, the following phenomenological conditions:

- The beads do not significantly swell or erode during drug release.
- The beads are spherical in shape.
- The drug is initially homogeneously distributed within the spheres.
- Perfect sink conditions are provided throughout the experiments.
- Mass transfer resistance due to liquid unstirred boundary layers at the surface of the spheres is negligible compared to mass transfer resistance due to diffusion within the systems.
- Drug dissolution is rapid and complete upon exposure to the release medium.
- Diffusion with time- and position-independent diffusion coefficients is the release rate-limiting mass transfer step.

These conditions were mathematically reduced to what was written above for a “sphere loaded initially with a homogenous concentration $C_{0}<C_{s}$ *C*_0_ < *C_s_* and maintained in a solution of constant concentration $C_{1}$.

#### 2.6.4. Release from Lipid Implants with Cylindrical Geometry

Guse et al. considered the release of lysozyme from a cylindrical-shaped implant based on glyceryl tripalmitate. Assuming that diffusion is the dominant drug release mechanism, based on Fick’s second law for cylindrical geometry [28,29], they considered that the release is the translation of the following phenomenological conditions:

- The implants do not significantly swell or erode during drug release.
- The implants are cylindrical in shape.
- Diffusional mass transport occurs in radial and axial direction, with the same diffusivities.
- The drug is initially homogeneously distributed within the implants.
- Perfect sink conditions are provided throughout the experiments.
- Mass transfer resistance due to liquid unstirred boundary layers at the surface of the implants is negligible compared to mass transfer resistance due to diffusion within the systems.
- Drug dissolution is rapid and complete upon exposure to the release medium.
- Diffusion with time- and position-independent diffusion coefficients is the release rate-limiting mass transfer step.

Using infinite series of an exponential function [30], the following solution was derived:

(23) $$\frac{M_{t}}{M_{\infty }}=1-\frac{32}{\pi^{2}}\sum \frac{1}{q_{n}^{2}}\exp(-\frac{q_{n}^{2}}{R^{2}}Dt)\sum_{p}^{}\frac{1}{{\left(2p+1\right)}^{2}}\exp\left(-\frac{{\left(2p+1\right)}^{2}\pi^{2}}{H^{2}}Dt\right).$$

The solution was found to describe well the release of lysozyme in the studied system.

### 2.7. Release Controlled by Transfer across Membranes, Considered as Coupled Interfaces: Release from Liposomes

**Figure 6:**
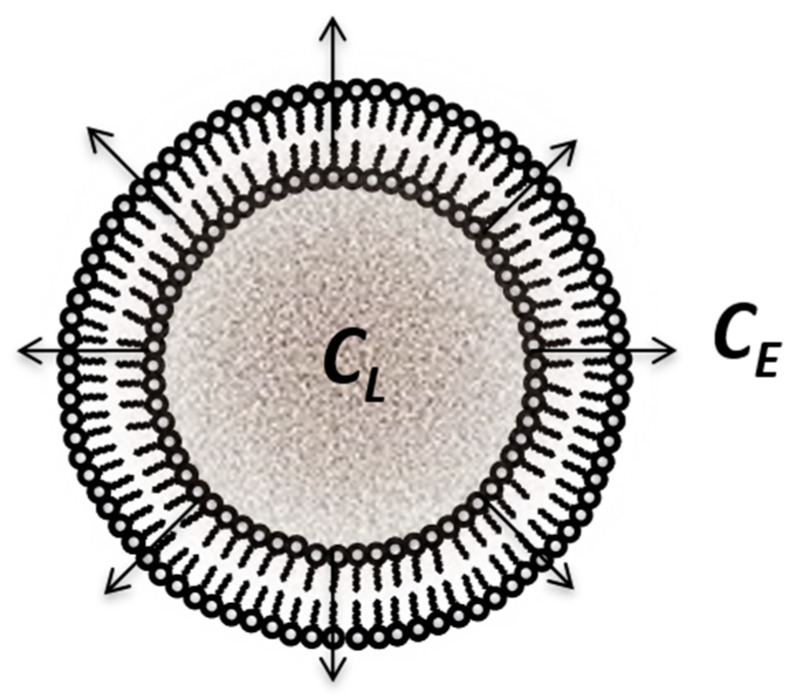
Release of active substance embedded in liposomes.

Coupled interfaces are very frequently met in transfer models as membranes for the control of release from lipid or solid microsystems. Since in such cases the interest moves from distribution inside membranes to transfer across membranes, the mathematical models concern concentrations in the interior and exterior of the membrane, as well as the content of membrane being neglected (Figure 6). Mathematical models are much more complex and solutions are obtained only for stationary conditions. 

A non-sink ultrafiltration method was developed to monitor liposomal release kinetics of the anticancer agent, topotecan. Mathematical modeling of the release data allowed simultaneous determination of drug permeability and interfacial binding to the bilayer [31].

A particular study concerned the release of local anesthetics from liposomes. Amphiphilic local anesthetics interact hydrophobically and electrostatically with lipid bilayers and modify their physicochemical properties, with the direct inhibition of membrane functions [32]. 

A complex model concerned the release from 100-nm liposomes, composed of hydrogenated soybean, phosphatidylcholine, poly(ethylene glycol)–distearoylphosphatidylethanolamine (PEG–DSPE), and cholesterol loaded with methylprednisolone, doxorubicin, or cisplatin [33,34]. 

The intra- and extra-liposomal domains were both considered to be well mixed, and it was assumed that encapsulated drugs may be released from liposomes via diffusion and/or liposome disintegration. The following equations were proposed:

(24) $$\frac{dM_{L}}{dt}=\frac{d\left(V_{L}C_{L}\right)}{dt}=V_{L}\frac{dC_{L}}{dt}+C_{L}\frac{dM_{L}}{dt},$$

where $M_{L}\left(t\right)$, $V_{L}\left(t\right)$ and $C_{L}\left(t\right)$ are the drug content (moles), volume, and average drug concentration of the entire liposome compartment, respectively. The terms $V_{L}\frac{dC_{L}}{dt}$ and $C_{L}\frac{dM_{L}}{dt}$ represent drug release via diffusion and due to volumetric changes in the liposome compartment, respectively. 

For calculation of $\frac{dC_{L}}{dt}$, the authors considered Fick’s first law, assuming the concentration gradient between the concentration of drug in liposomes $C_{L}$ and external concentration $C_{E}$ to be linear.

The solution of the equation is

(25) $$\frac{C_{L}(t)}{C_{L}(0)}=\frac{1}{r_{V}(t)}\left[1+\left(r_{V}(t)-1\right)\exp\left(-k_{0}r_{V}(t)\int_{0}^{t}\frac{dt}{r_{V}(t)-r_{L}(t)}\right)\right],$$

where $r_{V}(t)=\frac{V(t)}{V(0)}$, $r_{L}(t)=\frac{L(t)}{L(0)}$, and $k_{0}$ is a constant depending on permeability across the liposomal membrane and the radius of liposomes.

Taking into account the heterogeneity of the bilayer, Diamond and Katz [35] proposed a general model, considering local partition and diffusion coefficients at a depth *x* normal to the bilayer.

Xiang and Anderson further simplified the above model by assuming that permeability across a bilayer may be rate-limited to a distinct region (barrier domain) within the bilayer [36]. The barrier domain was shown to exhibit a chemical selectivity similar to that expected for the hydrocarbon chain region in liquid crystalline bilayers, although its properties vary somewhat with the lipid bilayer phase structure [37,38,39,40].

Sometimes, models for both the release and pharmacokinetics of drugs were attempted. For example, Kou et al. [41] proposed a model for the transfer of panciclovir embedded in liposomes applied on skin. The authors considered that, after the liposome was degraded, all of the encapsulated drug was exposed to the dermis tissue.

Mathematically, the hypotheses correspond to the diffusion, transfer, and degradation of liposomes containing penciclovir across the epidermis and dermis, as described by the following equations with initial and boundary conditions:

(26) $$\frac{\partial C_{L}}{\partial t}=D_{L}\frac{\partial^{2}C_{L}}{\partial x^{2}}+r_{A}\;\mathrm{and}\;r_{L}=-\frac{dC_{L}}{dt}=-k_{d}C_{L},$$

where $C_{L}$ is the concentration of the liposome, $D_{L}$ is the diffusivity of the liposome, $r_{L}$ represents the liposome degradation in the dermis (first-order degradation assumed), and $k_{d}$ is the degradation rate constant for the liposome.

For the diffusion of penciclovir in the epidermis and dermis, the degradation of liposome and the elimination of penciclovir are to be considered. The overall governing equation is

(27) $$\frac{\partial C_{P}}{\partial t}=D_{P}\frac{\partial^{2}C_{P}}{\partial x^{2}}+k_{d}C_{A}-k_{e}C_{B},$$

where $C_{P}$ denotes the concentration of penciclovir, $D_{P}$ is the diffusivity of penciclovir, and $k_{e}$ represents the first-order elimination constant of penciclovir.

Unfortunately, the equations cannot be solved and the model cannot be verified in terms of applicability in describing the evolution of skin and plasma level concentrations. 

## 3. Mechanistic and Empirical Models in Systems with Moving Boundaries

### 3.1. Matrix Systems

#### 3.1.1. Stefan’s Problem

Mathematical modeling in systems where a boundary is moving more quickly than diffusion could be considered as originating in Stefan’s papers around 1890, starting from the phenomenon of ice melting. He introduced a class of differential equations and boundary conditions which can be solved to give analytical solutions. Denoting the melted depth of the ice block as *s*(*t*), he introduced a new type of boundary condition, derived from the conservation of energy requirement.

(28) $$\frac{ds}{dt}=-\frac{\partial c}{\partial x}\left(s\left(t\right),t\right),\;t>0.$$

This is to underline that boundary terms have to be considered in a more large, mathematical meaning. For example, critical micelle concentration (CMC) is also a boundary. Diffusion coefficients are different below and above CMC. Time is also a dimension and has a finite or infinite boundary, such that initial conditions are, in the mathematical approach, a part of boundary conditions. Moving boundaries concern space, concentration, etc., which are variable during the diffusion process. More generally, we can discuss the problem of parameters in space; for example, the pH boundary was also studied as a parameter with discontinuous or critical evolution.

As a consequence, the first step in modeling the release of active substances from micro- and nano-drug carriers is the identification of phenomenological conditions, as well as of critical parameters and their formulation as boundary conditions. Furthermore, problems regarding the existence and uniqueness of solutions, and finding of the analytical solutions will appear. 

Modeling of the release from matrix systems, where the drug is dispersed or dissolved, involving moving boundaries, was reviewed recently, with focus on analytical solutions [42].

#### 3.1.2. Steady-State Higuchi’s Moving Boundary Model

**Figure 7:**
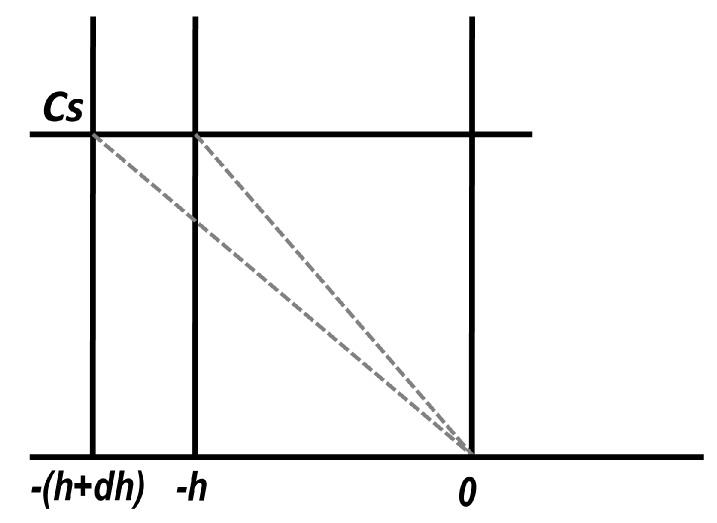
Higuchi’s moving boundary model inside solid and semisolid formulations.

In the pharmaceutical literature, these types of approaches started from the papers of Higuchi [43,44]. Well known and largely applied, Higuchi’s model considered that the solvent is gradually swelling the matrix, and the concentration gradient is linear, decreasing from the saturation concentration C_s_ at the interface with the core which was not attained by solvent, to concentration zero (sink conditions) at the matrix–dissolution medium interface (*x* = *h*) (Figure 7).

He obtained, for the released amount of drug, the following expression:

(29) $$M(t)=\sqrt{\left(2A-C_{s}\right)C_{s}Dt},$$

where *A* is the initial concentration of the drug in the matrix, and it is supposed that $A>>C_{s}$.

An exact analytical solution associated with the same phenomenological conditions, but replacing the hypothesis of linear gradient of concentration with $D\frac{\partial c}{\partial x}M(t)=\left(A-C_{s}\right)\frac{ds}{dt}$, was derived by Carslaw and Jaeger for melting and solidification in the Chapter XI (“Change of State”) of their book “Conduction of Heat in Solids” [2]. An analytical solution in terms of diffusion was obtained by Koizumi et al. in 1975 [45].

#### 3.1.3. Release from a Spherical Matrix

**Figure 8:**
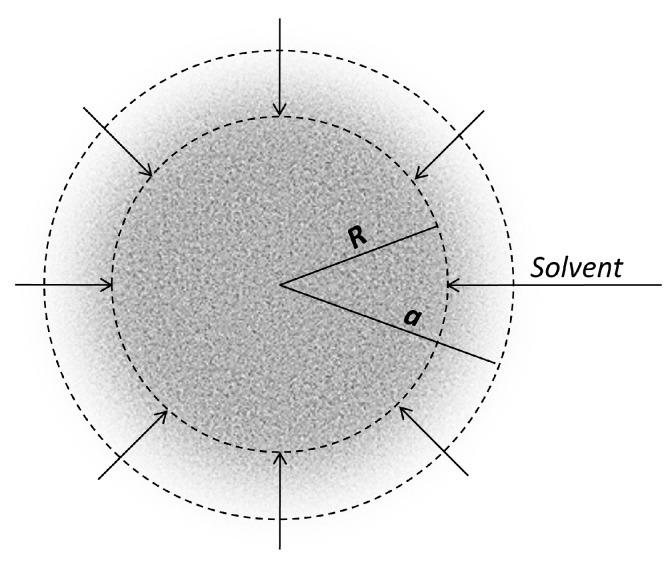
Higuchi’s model for release from a spherical tablet of radius *R*, in the condition of *a* moving solvent front.

Higuchi extended his initial method for release from a plane matrix to release from a spherical matrix [44] (Figure 8). 

The hypothesis concerning the linear gradient of concentration in the partially extracted matrix became

(30) $$C(r,t)=C_{s}\frac{R}{r}\frac{\left(a-r\right)}{\left(a-R\right)},$$

where *r* = *r*(*t*) is the coordinate, and *R*(*t*) and *a* are the radius of the “unreached core” and of the entire spherical particle, respectively.

The solution obtained by Higuchi for the time course of the amount released from a sphere was

(31) $$\frac{M_{t}}{M_{\infty }}=1-{\left(\frac{R}{a}\right)}^{3}+\frac{1}{2}\frac{C_{s}}{A}\left[2{\left(\frac{R}{a}\right)}^{3}-{\left(\frac{R}{a}\right)}^{2}-\left(\frac{R}{a}\right)\right].$$

Integration of the equation gives the relationship between the moving boundary interface position (*R*) and time (*t*) as follows:

(32) $$\frac{6C_{s}Dt}{Aa^{2}}=2{\left(\frac{R}{a}\right)}^{3}-3{\left(\frac{R}{a}\right)}^{2}+1-\frac{C_{s}}{A}\left[2{\left(\frac{R}{a}\right)}^{3}-4{\left(\frac{R}{a}\right)}^{2}+\left(\frac{R}{a}\right)+1+\ln\frac{R}{a}\right].$$

When $A>>C_{s}$, we can neglect $\frac{C_{s}}{A}$, and the equations take the approximate form

(33) $$\frac{M_{t}}{M_{\infty }}=1-{\left(\frac{R}{a}\right)}^{3},$$

and

(34) $$\frac{6C_{s}Dt}{Aa^{2}}=2{\left(\frac{R}{a}\right)}^{3}-3{\left(\frac{R}{a}\right)}^{2}+1.$$

Koizumi and Panosuk [46] obtained, in similar conditions to Higuchi, a solution in the form of a series, which, after some simplifications, had the following mathematical expression:

(35) $$M(t)=4\pi a^{2}\left[\sqrt{\left(2A-C_{s}\right)C_{s}Dt}+\frac{4C_{s}Dt}{9a}\left(\frac{C_{s}}{2A-C_{s}}-3\right)\right].$$

#### 3.1.4. Boundary Layer Effect

Since in the boundary layer at the interface between pharmaceutical formulation and release medium there is no stirring, this will act as a “resistance” to release.

Roseman and Higuchi [47] added this effect in the model and, for *A* >> *C_s_*, obtained the following equation:

(36) $$h^{2}+\frac{2D\delta h}{D_{a}}=\frac{2DC_{s}t}{A}\;\mathrm{and}\;M=Ah,$$

where δ is the thickness of the boundary layer, and *D_a_* is the diffusion coefficient in water.

At the beginning of release, when *h* << 1, it is possible to neglect *h*^2^, and the approximate solution results in

(37) $$M\left(t\right)=\frac{D_{a}C_{s}t}{\delta },$$

which means that *M*(*t*) is decreasing when *δ* increases.

As h increases, $h^{2}>>\frac{2D\delta h}{D_{a}}$ and $M\left(t\right)=\sqrt{2AC_{s}D_{a}t}$; thus, the effect of δ disappears.

### 3.2. Swellable Polymers

#### 3.2.1. Intrusion of Water into Matrix

Release of lysozyme, bovine serum albumin (BSA), alcohol dehydrogenase, and thyroglobulin proteins from monolithic triglyceride cylinders [48] was controlled by diffusion in the water intruding the lipid matrix. The model considered the solution of the diffusion equation in cylindrical coordinates obtained [49] with the boundary condition of homogeneous drug distribution at *t* = 0 (before exposure to the release medium) and perfect sink conditions:

(38) $$\frac{M_{t}}{M_{\infty }}=1-\frac{32}{\pi^{2}}\sum \frac{1}{q_{n}^{2}}\exp(-\frac{q_{n}^{2}}{R^{2}}Dt)\sum_{p}^{}\frac{1}{{\left(2p+1\right)}^{2}}\exp\left(-\frac{{\left(2p+1\right)}^{2}\pi^{2}}{H^{2}}Dt\right),$$

where *M_t_* and *M*_∞_ represent the absolute cumulative amounts of drug released at time *t* and infinite time, respectively; $q_{n}$ are the roots of the Bessel function of the first kind of zero order, and *R* and *H* denote the radius and height of the cylinder. The release strongly depended on the wettability of the material [50].

The same mechanism was also identified for the release from triglyceride microspheres [51,52]. When 0.1% Tween-80 was added to the release medium, the time to achieve 65 to 80% release decreased from 60 days to approximately 20 days. This could be explained by the fact that the surfactant improved the wetting of capillary walls, as well as the dissolution and release of active substance, phenomena also underlined by other authors [50,53].

#### 3.2.2. Swelling Component of Release from Polymers

The entering of liquid into the polymeric matrix promotes a series of complex processes and continuously modifies the diffusion conditions [30,54].

**Figure 9:**
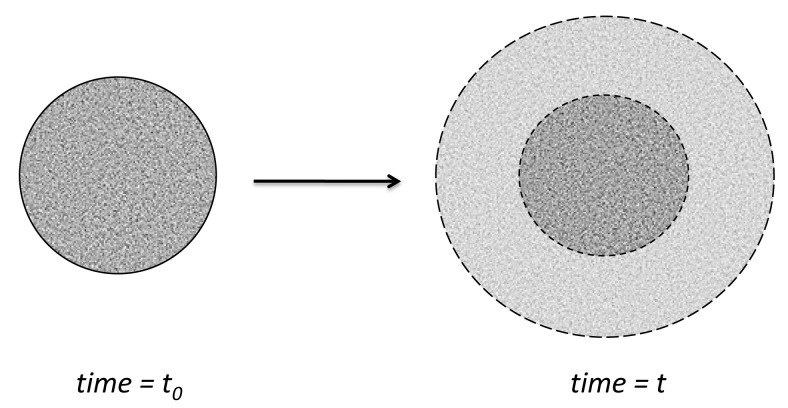
Swelling of a spherical polymer particle following the intrusion of solvent across the outer surface.

This is to consider primarily at least two different diffusion processes—that of the solvent inside the matrix and that of the drug into the penetrating liquid after its dissolution (Figure 9). 

The problem of solvent diffusion into the matrix is similar to sorption by a swelling sheet of thickness *l*. If the diffusion coefficient and concentration at the interfaces can be considered constant, the diffusion equation has an analytical solution [1] and the fraction “released” from the medium into the sheet is given by the following formula:

(39) $$\frac{M_{t}}{M_{\infty }}=1-\frac{8}{\pi^{2}}\sum_{p}^{}\frac{1}{{\left(2p+1\right)}^{2}}\exp\left(-\frac{{\left(2p+1\right)}^{2}\pi^{2}}{l^{2}}Dt\right).$$

Diffusion occurring concomitant with swelling was evaluated first by Hopfenber et al. in 1978 [55] and predictions were later attempted [56]; however, it soon became clear that this was too ambitious a task [57,58]. The diffusivity becomes concentration-dependent, increasing with both time and concentration of the liquid [59]. “Marginal” models, particularly for more symmetric particles, were further attempted [60,61,62]. 

Some formulations contain a mix of both soluble and insoluble polymers. Consequently, a significant swelling of the insoluble polymer occurs after partial dissolution of polymers and the drug, leading to the quick appearance of pores or even large cavities full of liquid through which the drug diffuses. Release from those systems was, as a rule, not satisfactory and was described as neither Higuchi nor Fickian behavior [63,64,65,66,67,68,69,70].

The most frequently applied model is the power law.

(40) $$M=kt^{n}.$$

Case II systems are characterized by *n =* 1 and Case I systems are characterized by n=1/2.

Non-Fickian systems lie between Case I and Case II, in that *n* takes an intermediate value between 1/2 and 1, and the curves change sigmoidally from one type to the other. Consequently, non-Fickian behavior needs two or more parameters to describe the interacting diffusion and relaxation inherent effects.

A simple expression of this observation can be heuristically written as the sum of the diffusion-controlled and relaxation-controlled drug delivery

(41) $$M_{t}/M_{\infty }=k_{1}t+k_{2}{\sqrt{t}}_{}.$$

The generalized expression $M_{t}/M_{\infty }=kt^{n}$, similar to that from Crank, was introduced in pharmaceutical literature in 1985 [58] and is known as the “Peppas equation”. The power law was used extensively to describe the first 60% of the release curves [71,72,73,74,75].

However, in the case of several hydroxypropyl methyl cellulose (HPMC)-based matrix tablets, it was demonstrated that the power law can describe the entire drug release profile [76]. Furthermore, the authors proposed a hypothesis for the theoretical justification of cases where the equation can really be extended to all release data, based on the non-classical diffusion of the solutes in HPMC matrices as disordered media. Simulations of the drug release in fractal matrices [77] or the percolation model [78] were used.

Sometimes, the rate of drug release follows neither the process of diffusion nor that of erosion; nevertheless, the equation could still be applied [79,80]. 

Some erodible polymers were developed specially for prolonged release of the active substance following its adhesion to gastric mucosa [81,82,83]; however, in these cases, the models are no longer applicable. 

For other geometries, different exponent values corresponding to different drug release mechanisms were proposed in literature [63,84,85,86].

### 3.3. Erodible Polymers

Release from erodible polymers was approached by many authors, with a great number of empiric and mechanistic models being developed. An excellent analysis and complete review of these models was performed by Arifin et al. [87].

The process of erosion of a polymeric matrix in a liquid happens only in part mechanically, with dissolution being, in most cases, the main process in its initiation and evolution. The liquid diffuses into the polymer and locally dissolves both the drug and the polymer. Thus, the surface of the dosage form becomes a moving boundary. 

The simplest model assumes that the rate of erosion of a film of thickness *l* remains constant during the process ($v=\frac{dl}{dt}$), and the initial concentration of the drug is uniform in the dosage form.

#### 3.3.1. Kinetics of Release from a Sheet of Thickness 2*l*

In this case, the thickness of the sheet depends on time, and follows the equation

(42) $$l_{t}=l_{0}-vt,$$

where $l_{0}$ is the initial thickness and $l_{t}$ is the thickness at time *t*. Erosion ends at time $t_{f}=\frac{l_{0}}{v}$. 

Replacing v in the expression of $l_{t}$ results in

(43) $$l_{t}=l_{0}(1-\frac{t}{t_{f}}).$$

Since the volume of the sheet is proportional to time, the amount of drug released at time *t* is also proportional to time and, upon combining the two expressions, it can be written in the form

(44) $$\frac{M_{t}}{M_{\infty }}=\frac{t}{t_{f}}.$$

#### 3.3.2. Kinetics of Release from a Sphere of Initial Radius $r_{0}$

Similar to the above case, for a sphere with radius $r_{0}$,

(45) $$r_{t}=r_{0}-vt=r_{0}(1-\frac{t}{t_{f}}),$$

and

(46) $$\frac{M_{t}}{M_{\infty }}=1-{\left(1-\frac{t}{t_{f}}\right)}^{3}.$$

#### 3.3.3. Kinetics of Release from a Cylinder of Radius $r_{0}$ and Height 2$h_{0}$

In the case of a cylinder, it is necessary to consider the decrease in size following the erosion of length *h* and radius *r*.

(47) $$\frac{M_{t}}{M_{\infty }}=1-{\left(1-\frac{vt}{r}\right)}^{2}{\left(1-\frac{vt}{h}\right)}^{2}.$$

Release from bioerodible polymers is highly complex, since we have a continuous change in local conditions due to the coexistence of diffusion, chemical reactions, moving boundaries, volume changes, appearance of oligomers and monomers, pores, holes, etc., and mathematical modeling is consequently more difficult [88,89,90].

**Figure 10:**
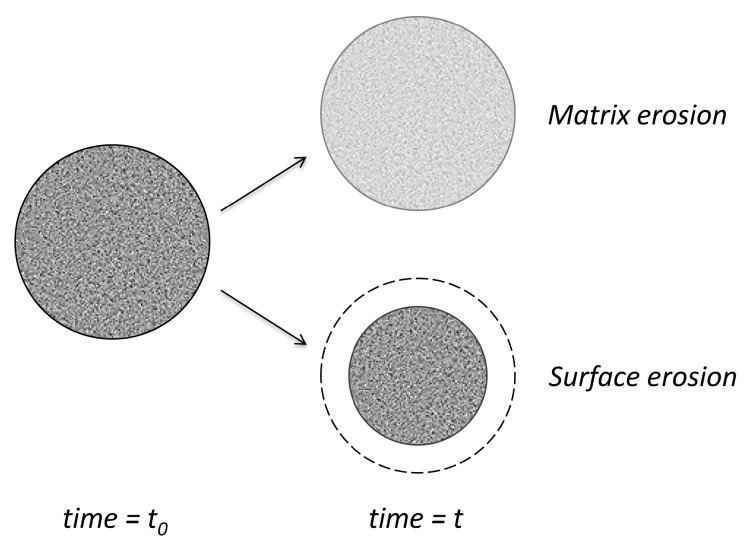
Marginal-type erosion models.

Depending on the rate of water diffusion in polymers and the rate of degradation, erosion evolution concerns mainly the surface or bulk structure [91] (Figure 10). If degradation is much slower than the diffusion of water and, therefore, the limiting step, the changes will be homogeneously distributed in the bulk of matrix. If degradation is more rapid, the surface erosion will be the main effect. For example, polyanhydrides are more reactive and, consequently, the surface erosion is predominant. For polylactides (PLA), degradation leads to rather bulk erosion. In fact, in all cases, both types of degradation coexist in different proportions.

Most of the polymers used in practice are biodegradable in order to avoid problems connected with elimination of non-biodegradable, big molecules from a living body. Elimination decreases upon increasing the size of particles [92,93]. 

Some polymers, for instance, polydimethylsiloxanes or polyurethanes, are biodegradable; however, since the degradation time is far greater than the time of active substance release, from the point of view of release kinetics, they are considered as “non-biodegradable”. They are long circulating systems that produce particular in vivo pharmacokinetics (e.g., residence time, distribution, clearance, half-life, etc.), providing a prolonged effect of the respective drugs. Such an example is that of micelles. Consequently, micelles of block copolymers including amphiphilic and hydrophobic surfactants were developed as carriers for poorly soluble drugs [94].

Initiation of erosion implies the need to additionally consider the hydrolysis following the penetration of water molecules, which leads to changes in all polymer characteristics; pores, holes, oligomers, and even monomers appear. 

#### 3.3.4. Empirical Surface Erosion Models

Empirical models are global characterizations of the release processes without taking into consideration all processes involved during release. Such an approach is clearly a much easier task, since, in a chain of processes, the global rate is given by the slowest process, with this approach being, in many cases, the most appropriate. The models are tested statistically. The disadvantage of empiric models is the fact that simulations and predictions are less performant than in the case of mechanistic, complex models, based on the physicochemical picture of the evolution of phenomena.

The Hopfenberg model [95] considered that dissolution, swelling, and polymer chain scission can be described by as a final zero-order process and established the following formula:

(48) $$\frac{M_{t}}{M_{\infty }}=1-{\left(1-\frac{k_{0}t}{C_{0}a}\right)}^{n},$$

where *n* = 3, 2, and 1 for spheres, cylinders, and thin films, respectively; *a* is the radius of the sphere or cylinder or half thickness of thin film, *C*_0_ is the initial drug concentration in the system, and *k*_0_ is the equilibrium rate constant. 

It is to note that the equation proved to be applicable for surface-eroding systems. The problem is that *a* is not constant in time. 

The formula is a generalization of the result found by Hixson–Crowell [96], starting from the fact that, for spherical particles, volume is proportional to *a*^3^ and area is proportional to *a*^2^. El-Arini and Leuenberger [97] modified the Hopfenberg model by accounting for the lag time ($t_{lag}$) before the release process to start.

(49) $$\frac{M_{t}}{M_{\infty }}=1-{\left(1-\frac{k_{0}(t-t_{lag})}{C_{0}a}\right)}^{3}.$$

A more detailed model, based on the same assumptions of a zero-order reaction at the surface of the polymer, for example, detachment of monomers, following their diffusion in the release medium, was performed by Cooney [98]. 

With the assumption of constant concentration difference existing between the surface and the dissolution medium (Δ*C*), and with the assumption that the surface-eroding matrix has a uniform drug distribution, we can obtain the following relationship for the release fraction:

(50) $$\frac{M_{t}}{M_{\infty }}=1-{\left(1-\frac{k_{ero}\Delta C\;t}{\rho_{s}a}\right)}^{3},$$

where $\rho_{s}$ is the density of drug in the matrix. The equation is similar to that of Hopfenberg, with the sole difference being that the concentration gradient (which is constant) appears explicitly. 

#### 3.3.5. Mechanistic Surface Erosion Models

Heller and Baker [99] considered a more in-depth analysis of the permeability factor in Higuchi’s formula.

(51) $$\frac{dM_{t}}{dt}=\frac{A}{2}\sqrt{\frac{2PC_{0}}{t}},$$

where permeability *P* is no longer constant but is a function of the number *Z* of pores created following erosion. 

Here, it is considered that cleavage follows first-order kinetics.

(52) $$\frac{dZ}{dt}=k(N-Z),$$

where *N* is the initial number of bounds.

The solution is an exponential and, considering that $\frac{P}{P_{0}}=\frac{N}{N-Z}$, the Higuchi formula becomes

(53) $$\frac{dM_{t}}{dt}=\frac{A}{2}\sqrt{\frac{2P_{0}e^{Kt}C_{0}}{t}}.$$

Harland et al. [100], as well as Kosmidis et al. [101], built a model for bulk erosion in conditions of both infinite and finite boundary conditions. They also took into consideration diffusion into fine pores [102].

Their model for diffusion from microspheres was based on the following equation:

(54) $$\frac{\partial c}{\partial t}=D_{e}\left(\frac{\partial^{2}c}{\partial r^{2}}+\frac{2}{r}\frac{\partial c}{\partial r}\right)+k\left(\varepsilon C_{s}-C\right),$$

where *C* and *D_e_* are the drug concentration and effective diffusivity in liquid-filled pores, respectively; *k* is the drug dissolution rate constant, *ε* is the porosity of the polymer matrix, and $\varepsilon C_{s}$ is the saturation concentration in the solution filling the pores.

In the end, two analytical solutions were obtained, one appropriate for early diffusion and the other for later diffusion. This is to underline that the solution of a differential equation with usual initial and boundary conditions is unique. Different expressions of the solution can be found using different methods, but they cannot be considered as different functions. 

### 3.4. Complex, Multiparameter Release Models

#### 3.4.1. Concomitant Depolymerization, Erosion, and Diffusion

When degradation occurs, the matrix will become heterogeneous in terms of the distribution of the molecular weight of the polymer chains and pores created on the surface. This will induce further changes in the diffusion coefficient of the drug, which will become a function of both time and space.

Models of release in these conditions were studied by Himmelstein and co-workers. A model for thin-film geometries to describe the drug release from surface-erodible polymer matrices was developed [103]. 

Thombre and Himmelstein [104,105] developed a mathematical model for simultaneous transport reaction and delivery from a catalyzed bioerodible polymer matrix of polyorthoester. They considered the effect of the degradation process on the diffusion coefficient, with the diffusivity of all species (water, acid generator, acid, and drug) related to the extent of polymer hydrolysis according to the following expression:

(55) $$D_{i}^{}=D_{i,0}e^{\frac{\alpha (C_{D,0}-C_{D})}{C_{D}}},$$

where $D_{i}$ is the diffusion coefficient of species *i* when the polymer is hydrolyzed, *C* is the concentration of species *i* at time zero and *t* respectively, and alpha is a constant.

Other models took into consideration the increase in diffusion coefficients and drug release following a decrease in the molecular weight of polymers [106]. The authors considered that the PGLA matrix suffers degradation following first-order kinetics, and that drug diffusion is inversely proportional to the molecular weight.

(56) $$\frac{D_{e}}{D_{0}}=\frac{M_{w,0}}{M_{e}}\Rightarrow D_{e}=D_{0}e^{k_{\deg r}t}.$$

By introducing the result to the Higuchi formula, the equation becomes

(57) $$\frac{dM_{t}}{dt}=A\sqrt{\frac{2C_{0}C_{s}D_{0}(e^{k_{\deg r}t}-1)}{t}}.$$

This method and its result are similar to the results of Heller.

Lee [107,108] suggested that both swelling and mass erosion could be modeled using the same type of diffusion equations. He considered time-dependent diffusion coefficients defined as

(58) $$D_{t}=D_{i}-\left(D_{\infty }^{}-D_{i}^{}\right)\left(1-e^{-kt}\right).$$

Raman et al. [109] used the diffusion model for spherical geometry with diffusivity dependence on molecular weight to explain the piroxicam release from bulk-erosion poly(lactide-*co*-glycolide) (PLG) microspheres.

Molecular weight was considered to decrease exponentially like in the above models, but a time lag before the erosion of matrix started was additionally considered. 

He et al. [110] considered an exponential decrease in molecular weight, a time lag, and the time to maximum erosion rate, and obtained the following formula for the released fraction:

(59) $$\frac{M_{t}}{M_{\infty }}=4\sqrt{\frac{D_{e}t}{\pi r^{2}}}-3\frac{D_{e}t}{r^{2}}+F_{E}\left[\frac{e^{k_{\deg r}\left(t_{}-t_{max}\right)}}{1+e^{k_{\deg r}\left(t_{}-t_{max}\right)}}\right].$$

Depending on of the sign of the difference $t-t_{max}$ and the value of the last fraction, it is possible to explain the “S” shape of erosion curves, which is predicted by the Zhang model [111], including an initial “burst” and an intermediate rapid release.

Similar models were proposed by Siepmann [112] for experimental data concerning the release of 5-fluorouracil concomitant with bulk erosion of PGLA microspheres, and by Wada [113] for explaining the release of aclarubicin from PLA-based microspheres. Siepmann [114] also took into account the autocatalytic effect to explain the release of lidocaine from PLGA microspheres. 

#### 3.4.2. Monte Carlo Simulation Models

Zygourakis [115,116] considered the dissolution of drug and polymer and the lifetimes of drug, polymer, filler, or pore as pixels in two-dimensional grids. The lifetime of a pixel started to decrease upon contact with the solvent. The dissolution rates of the drug and polymer were defined, starting from the first law of diffusion, as

(60) $$\frac{dV_{d}}{dt}=\frac{k_{d}S_{d}\left(C_{d,s}-C_{d,b}\right)}{\delta_{d}},$$

and

(61) $$\frac{dV_{p}}{dt}=\frac{k_{p}S_{p}\left(C_{p,s}-C_{p,b}\right)}{\delta_{p}},$$

where the letters *p* and *d* refer to the polymer and drug, while *b* is the bulk, *S* is the surface, s is the saturation, *V* is the volume, and δ is the thickness of the limit layer. When diffusion is negligible, these formulas became the usual dissolution equations.

Both Monte Carlo simulation-based polymer degradation and diffusional mass transfer processes were taken into account in the models developed by Gopeferich et al. [115,116,117,118,119,120,121].

Macheras, in cooperation with a team of physicists, developed a complex theory for the study of the escape of particles from devices of fractal geometry [122]. The application of the theory in actual pharmaceutical finite systems is a much more difficult mathematical problem than analysis in infinite systems. Particles were considered randomly placed on the open sites of a matrix, from which they look to escape following random walks. A particle may stay immobile with a probability *q*, or move at a new randomly chosen neighboring site with probability *1* − *q*. When the particle is continuously moving (*q* = 0), the equation characterizing diffusion is obtained [77,123,124,125], but the result, as presented above, is much more general, in the frame of heat transfer, fluid mechanics, quantum theory, etc.

The case q≠0 allowed the authors to simulate diffusion processes with different diffusion coefficients. 

Monte Carlo simulation led to a differential equation of the form

(62) $$\frac{dN}{dt}=-af(t)N,$$

where a is a proportionality constant, and *f*(*t*)*N* denotes the number of particles that are able to reach an exit in a time interval *dt*. Assuming that *f*(*t*) is of the “fractal kinetics” form $f(t)\approx t^{-m}$, they found the Weibull function as a solution for the number of particles remaining inside the lattice.

(63) $$N(t)=N_{0}\exp(-at^{b}).$$

As presented later in the paper, the same team suggested that the Weibull function is a theoretical base for almost all release kinetics in heterogeneous matrices [126]. Monte Carlo simulations of the release process allowed to evaluate how Weibull coefficients *a* and *b* depend on the diffusion coefficient in the case of matrices with high- and low-diffusivity areas. It was obtained that the exponent *a* is smaller for low diffusion coefficients and the relationship between *a* and *q* is quasi-linear [122].

#### 3.4.3. Artificial Neural Network Models

**Figure 11:**
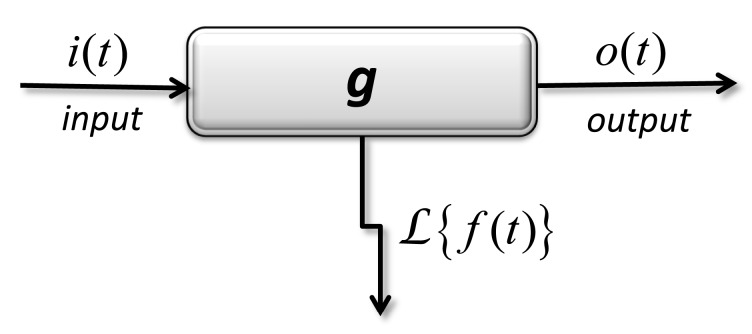
Black-box model of transfer (weighting) function, defined in the space of image functions obtained after the application of an integral transformation.

Artificial neural networks were also used to model drug delivery [127,128,129,130]. Tools coming from the theory of dynamic systems, as well as from pharmacokinetics are shown in Figure 11, where input *I* = *L*(*i*), output *O* = *L*(*o*), and transfer *G* = *L*(*f*). 

The application of Laplace transform leads to a definition of the transfer function *G* between the transformations of input and output functions.

*I* (*p*) ⇒ *G* ⇒ *O*(*p*) by relation *G I* (*p*) = *O*(*p*). (64)

The neural networks attempt to simulate some of the neurological processing abilities of the human brain. Specific names are “neurons”, connected by synapses. Input neurons are, for example, characteristics of formulations such as drug content, compression force, or composition in terms of excipients. In the case of release models, output neurons represent the performance of the formulation. 

The model can be further applied to correlate the release kinetics with pharmacokinetics as a parameter in vivo. The combination of the two “correlations” provides a correlation between formulation properties and in vivo performance [131].

The estimated weighting function can be used to “train” the network, i.e., to define, following successive approximations, the optimal equations and weights allowing for the calculation of the output values based on the input values in order to make quantitative predictions. This type of analysis was performed by Takahara et al. [128] to simulate the effects of the amounts of microcrystalline cellulose and hydroxypropyl methylcellulose, as well as the effect of the compression pressure used to prepare trapidil matrix tablets on the resulting drug release kinetics. Ibric et al. [132] studied acetylsalicylic acid release from Eudragit RS-based matrix tablets, whereas Ghaffari et al. [133] applied neural network algorithms for modeling theophylline release from coated pellets.

## 4. Release Models Based on Fick’s First Law

Fick’s first law concerning the flux of substances *J* across virtual interfaces in homogenous solutions is given by the following formula:

(65) $$\frac{1}{A}\frac{dm}{dt}=J=-D\frac{\partial c}{\partial x},$$

where *m* is the transferred mass, *A* is the area, *D* is the diffusion coefficient, and *c* is the concentration.

If we extrapolate the transfer at virtual interfaces in solutions toward the transfer between actual interfaces of pharmaceutical formulations with the release medium, Fick’s equation can be used to formally derive mathematical models usually considered for the analysis of data in the case of diffusion-controlled release processes.

### 4.1. Noyes–Whitney Model

In the “receptor solution” at the frontier with the pharmaceutical formulation appears the “limit” or “stationary” layer of thickness *h*, which is not affected by convection currents in the fluid. Let us consider that, in this limit layer, the concentration gradient is linear. It is natural to accept that the concentration of active substance in the immediate neighborhood of pharmaceutical formulation is equal to its maximum value $C_{s}$ (denoted frequently by *S*) given by its solubility.

(66) $$\frac{\partial c}{\partial x}=\frac{c_{h}-S}{h}.$$

Replacing the expression of concentration gradient, Fick’s equation is transformed into

(67) $$\frac{1}{A}\frac{dm}{dt}=-D\frac{\left(c_{h}-S\right)}{h}.$$

This expression is known as the Nernst–Brunner equation [134], established more than one hundred years ago.

This equation is theoretically very attractive; however, in practice, we cannot experimentally measure the thickness *h* of the limit layer, nor can we measure the diffusion coefficient D in proximity of the interface. The area of the interface *A* is also rather difficult to estimate and is not constant over time. 

If, for some time interval, we can assume that the expression $A\cdot \frac{D}{h}$ is constant, a simpler law is obtained.

(68) $$\frac{dc}{dt}=k(c_{s}-c_{h}),$$

which was experimentally established by Noyes and Whitney a long time ago [135]. This differential equation can easily be solved with initial condition $c_{h}(0)=0$ and the implicit solution is then obtained.

(69) $$-\ln(1-\frac{c_{h}}{c_{s}})=kt.$$

If we can accept that, beyond the limit layer, homogenization is rapid and concentration is the same ($c_{h}$) across the dissolution media, the representation of $-\ln(1-\frac{c_{h}}{S})$ versus time leads to an approximately straight line. Such a linear dependence can be considered as evidence that the process follows the Noyes–Whitney law. We can observe that the Noyes–Whitney law is a model with a single parameter, *k*. 

### 4.2. “Empirical” Extensions

If release is made in a medium of constant volume *V* and if we amplify with *V* the ratio $\frac{c_{h}}{c_{s}}$, the new fraction $\frac{V\cdot c_{h}}{V\cdot c_{s}}$ can be written as $\frac{m(t)}{m_{\infty }}$, where $m_{\infty }$ is considered the maximum quantity of active substance which can be released in limited solubility restrictions, whereby the solution of the equation can be written in the following alternative form:

(70) $$-\ln(1-\frac{m(t)}{m_{\infty }})=kt.$$

Most frequently, in dissolution tests, an increase in volume until $c_{h}\approx 0$ is attempted, often referred to as “sink conditions”. In such cases, the process ends when the entire quantity or alternatively all the “fraction available for release” is released. Consequently, it is easy to write $m_{\infty }$, but it is sometimes quite difficult to define it.

In order to obtain a “more flexible” model, we can replace *t* by a power term $t^{\beta }$.

(71) $$-\ln(1-\frac{m(t)}{m_{\infty }})=kt^{\beta }\;\mathrm{and}\;1-\frac{m(t)}{m_{\infty }}=e^{kt^{\beta }}.$$

Thus, a more general model was obtained, but the model is “empirical” since there is no theoretical justification for making the $t\to t^{\beta }$ substitution. Such a model was applied first in describing dissolution data by Langenbucher [136].

The above equation can be rewritten in the form

(72) $$\frac{m(t)}{m_{\infty }}=1-e^{-\alpha t^{\beta }},$$

which mathematically represents the cumulative Weibull distribution and, consequently, we can think to the interpretation of α as a “scale factor” and β as a “shape factor” in the Weibull survival distribution.

A linear dependence can be obtained by transforming the previous equation. A second-order logarithm expression is applied, and the following mathematical equation is obtained:

(73) ln(−ln(1−r))=lnα+βln(t),

where $r=\frac{m(t)}{m_{\infty }}$.

Consequently, if the graphical representation of ln(−ln(1−r)) versus ln(t) appears to be a straight line, we can assume a Weibull empirical dependence between *r* and *t*.

This function was and still remains most frequently applied to the analysis of dissolution and release studies involving nanoparticulate drug systems: nano- and micro-capsules [137,138], nanosuspensions [139], nanosized zeolits [140], PLGA nanoparticles [141], inorganic nanoparticles [142,143], solid lipid nanoparticles (SLN), and nano-structured lipid carrier (NLC) [144], as well as different liposomal formulations [145,146,147,148].

From analysis of the experimental data concerning the release of diltiazem and diclofenac [123], Papadopoulou et al. concluded that β is an indicator of the mechanism of transport of the drug through the polymer matrix; β≤0.75 indicates Fickian diffusion, while a combined mechanism (Fickian diffusion and swelling-controlled transport) is associated with β values in the range 0.75<β<1. For values of β higher than 1, the drug transport follows a complex release mechanism [126]. 

We make the observation that the usual classification of the Weibull model as “empirical” is a superficial analysis. The Weibull distribution function is the simplest distribution applicable to a multi-step chain, for example, survival in cancer. The probability that a patient is alive at moment *x* is conditioned by his or her survival at all *n* previous moments $(1-P_{n})={\left(1-P_{}\right)}^{n}$ and can be written in the form $e^{-n\varphi (x)}$.

The most common criticism is that this distribution function has no theoretical basis. However, as said Weibull, this objection applies practically to all other distribution functions related to real data from the natural or biological field, where, in almost all cases, the complexity is so high that it is utterly hopeless to find a theoretical base.

Particularly, in the case of release kinetics, when drug release takes place at interfaces, the hypothesis of homogeneous conditions is no longer valid for the entire course of the process. Macheras [149] proposed a replacement of the “reaction constant” in the Noyes–Whitney equation (*k*) with a function of time $k=k_{1}t^{-h}$.

After integration, the amount of released drug is described by the Weibull equation.

(74) $$M(t)=1-\exp\left(-\frac{k_{1}}{1-h}t^{1-h}\right).$$

Consequently, the “empirical” attribute of Weibull and Peppas models does not refer to empirical models, but refers to the fact that the models are fitted to the experimental data without examination of the conditions that were considered in the deduction of the mathematical expressions of respective models.

A further degeneration of the Weibull model for small $\alpha t^{\beta }$ values can be considered.

(75) $$\frac{M_{t}}{M_{\infty }}=1-e^{-\alpha t^{\beta }}\approx 1-(1+\alpha t^{\beta })\approx \alpha t^{\beta }.$$

Thus, the above discussed Peppas law is obtained.

The Higuchi law appears, therefore, to be a particular case of Peppas law, for β=1/2. 

### 4.3. Applications of “Empirical” Models in Describing Release from Micro- and Nanostructured Carriers

All the above deductions are mainly empirical and formal; however, this is to underline that mechanistic models are difficult to understand due to mathematically complex aspects and they are difficult to apply since they require a large amount of experimental data. These are the main reasons for the fact that the application of empiric models is more widespread than the application of mechanistic models. A number of papers where data are analyzed using empirical rather than mechanistic models are presented below.

On other hand, as presented above, application of so-called “empirical” but in fact not empirical models allows, following a more in-depth analysis [126] of the β coefficient, an estimation of the drug release mechanisms. 

As a general rule in the selection of models, the simpler ones are preferred to the more complex ones, since they are more stable to variations of the experimental data. For example, the Higuchi model, a one-parameter model, is often preferred to the power law and Weibull models, which depend on two parameters, in spite of the fact that it cannot describe all experimental data.

As presented in the tables below, square-root and power models were analyzed and were considered “good” in almost all experimental cases. Since the Weibull model has two parameters and since power models can be considered degenerated Weibull models, it is clear that Weibull can better fit the experimental data in all cited examples. Whatever the reasons to avoid the Weibull model (and maybe the main reason concerns the “empirical” label), in many cases, the analysis of obtained parameters can give essential information about the release mechanisms. For all the above models, we can see that decreasing the size to micro- and nano-domains causes many of the concepts from the continuous, homogeneous phases to become questionable, as we enter more and more into a fractal space. The concept of fractal geometry can be applied to describe the complexity of the heterogeneous nature of drug release processes both in vitro and in vivo [150,151,152,153,154].

#### 4.3.1. Micro-Sized Polymeric Carriers

As previously presented in the paper, release from a polymeric matrix is a complex process implying numerous phenomena. In the case of microsystems, there are many additional difficulties in modeling drug release data, as there is a great diversity in the physical form of formulations with respect to size, shape, arrangement of the sheets, etc. Diversity of active substances is great, and their physicochemical properties are modified following their combination with excipients in the engineering of micro and nano formulations. There are also problems in translating kinetics of drug release from “micro” products of homogeneous geometrical space to various irregular systems [155]. Consequently, as can be seen in Table 1, the fitting of release with solutions of empirical models is the rule rather than the exception.

#### 4.3.2. Nano-Sized Polymeric Carriers

The release kinetics of active substances from nano-sized polymeric carriers following their small size in many cases can no longer be adequately described by models used in the case of micro-sized carriers. Some reasons for failure could be as follows:

- the release models developed for transfer across plane surfaces are no longer applicable;
- their curvature implies specific properties, primarily high free energy and aggregation tendency;
- continuum models lack the ability to describe the kinetics of drug release as the concentration of the drug in the nanosystems fluctuates and the notion of concentration profile becomes meaningless.

In terms of their interaction with biological fluids, nanosystems tend to be stable (no degradation and/or dissolution in blood). Non-biodegradability is relative to the time scale associated with the drug release process. In fact, many studies of nanocarriers revealed that the encapsulated drug is completely released before polymer degradation occurs. Consequently, the release kinetics from nanocarrieres is even more frequently based on fitting experimental data with solutions of empirical models (Table 2).

#### 4.3.3. Liquid Crystals

Liquid crystals are formulations at the frontier between continuous and multi-particulate structures, with their essential property being the appearance of fluid ordered domains, appearing essentially as a consequence of including active substances in surfactant–cosurfactant structures. The most widely used liquid crystal system appears to be cubosomes. Research of cubosomes as a drug delivery system involved oral [176], intravitreal [177], and subcutaneous [178] routes of administration [179,180]. A novel vehicle based on cubosomes was used as an ophthalmic drug delivery system for flurbiprofen (FB) in order to reduce ocular irritancy and improve bioavailability [181]. Transdermal enhancing effect of cubosomes was reported by some researchers [182]; this effect might be due to the structural organization of cubosomes, which is similar to that found in biomembranes [183,184]. 

Liquid crystals present many advantages for drug delivery, including their ability to incorporate both hydrophilic [185] and hydrophobic drugs [182] and their possibility to function as sustained-release delivery systems [186].

What is surprising is that, in almost all cases, their release kinetics are fitted by solutions of simple empirical models (Table 3). The usual proposed model is the Higuchi square-root law [187].

A more in-depth analysis revealed that cubosomes should be classified as a burst release delivery system, whereby drug is released by diffusion from the cubic phase matrix and the critical factor is represented by the nature of surfactants. As the HLB of additives in matrix increases, release is shifted from anomalous (non-Fickian) diffusion and/or partially erosion-controlled release to Fickian diffusion. Initial lag time was observed for drug released from matrices with additives of HLB 1.5, 3, 4, and 5. Thus, the incorporation of additives of different HLBs led to a modification of molecular packing, which significantly affected the drug release pattern [176,177,178,185,188,189,190,191]. 

#### 4.3.4. Liposomes

Although liposomes, following their spherical symmetry and relatively simple boundary conditions, are good candidates for mechanistic models, results that could be appropriately described by empiric models were also published.

Oezyazici et al. [199] investigated metronidazole release from different types of lipid matrix tablets and found Higuchi’s model as being appropriate. The same model was proposed for describing the release of safingol from liposomes prepared with distearoylphosphatidylcholine and cholesterol.

First-order models, and the Higuchi or Hixson–Crowell equations could appropriately fit the experimentally determined drug release kinetics from different liposomal formulations [189,200]. Weibull and power-law models were used for describing the release of indomethacin liposomes based on dipalmytolphosphatidylcholine and poly(2-methyl-2-oxazoline)-*g*-poly(2-phenyl-2-oxazoline [146]. Release of baicalin from liposomes based on Tween-80, phospholipon^®^ 90H, and citric acid in phosphate-buffered saline (pH 7.4, PBS) using a dialysis technique was best described by the Weibull model [147].

#### 4.3.5. Solid Lipid Nanoparticles and Lipid Dosage Forms

The main lipid dosage forms are lipid microparticles and spherical beads. The most frequently applied semiempirical model was the “power law” [189,200]. More detailed examples in this respect are presented in Table 4. 

A plot of “1 − (1 − *r*)^0.5^” versus the square root of time for in vitro release of interferon a (IFNa) from lipid cylindrical matrices based on tetraglycerol tripalmitate (squares), tetraglycerol monopalmitate (filled triangles), tetraglycerol dipalmitate, tetraglyerol distearate, or tetraglyerol monostearate led to a linear dependence [201].

### 4.4. Selection of the Mathematical Release Model

In virtually all cases of supramolecular systems, there is no possibility to elaborate a mechanistic model, i.e., a model taking simultaneously into account the structure and properties of the system, as well as those of the drug and their interactions. 

For that reason, as presented above, empirical and semiempirical models are usually attempted in order to fit the experimental data. In spite of the fact that phenomenological conditions for the respective system are not verified, if the fitting of experimental data “works well”, the model is considered applicable. To illustrate this widespread approach, we chose three papers published in the last year, concerning the release from cubosomes, where an appropriate fitting of experimental data using empirical models can be obtained in virtually all cases.

In a paper concerning comparative in vitro and in vivo studies on glycerol monooleate and phytantriol-based cubosomes containing oridonin [206], the zero- and first-order models, as well as Higuchi and Weibull equations, were tested. A linear relationship was established between the release rate and the square root of time for both cubosome formulations, indicating that the release kinetics fit Higuchi’s equation and were controlled by drug diffusion. The criterion for this selection was the correlation coefficient *R*^2^ (0.9924 and 0.9972).

Another study [207] presented the development and characterization of novel small self-assembled resveratrol-bearing cubosomes and hexosomes. To analyze the release kinetics of resveratrol from those formulations, the obtained data were fitted into zero-order, first-order, Higuchi, and Korsmeyer–Peppas models. The agreement of fit for most formulations was achieved with the Higuchi kinetic model (*R*^2^ ≥ 0.9724). 

Such examples can be multiplied since practically all release studies were analyzed similarly.

A much more in-depth analysis was proposed in 2019 [208]. Authors systematically studied the release kinetics of fluorescein from colloidal liquid crystals obtained from monoglyceride and different non-ionic surfactants.

The appropriate mathematical model and the hierarchy of the performances of the linear, Noyes–Whitney, square-root, Siepman–Peppas, and Weibull models applied to the release experiments was attempted. 

The essential difference from previous papers was the application of informatics criteria (Akaike information criterion (*AIC*) [209], Schwarz criterion (*SC*) [210]) and also the Fisher test to the correlation coefficient.

The Akaike information criterion (*AIC*) [209] and Schwarz criterion (*SC*) [210] are based on the addition of statistical errors corrected by a penalty function, proportional to the number of parameters (p) evaluated in the following models:

(76) AIC=NlnSS+2p,

(77) SC=NlnSS+plnN,

where *N* represents the number of point data, and squared errors *SS* represent the sum of squared deviations of a model with a set of p parameters, calculated according to the following equation:

(78) $$SS=\sum_{i=1}^{n}{\left(y_{i}^{exp}-y_{i}^{calc}\right)}^{2}.$$

The model equations having the lowest *AIC* or *SC* were selected for the evaluation of the time course plots.

Fisher (*F*) test criterion permits comparing a simple model having *q* parameters with a complex model having supplementary *k* parameters, with *p = q + k* using the *F* ratio, according to the following equation:

(79) $$F=\frac{SS_{q}-SS_{p}}{SS_{p}}\frac{df_{p}}{df_{q}-df_{p}},$$

where $SS_{q}$ is the sum of standard errors for the selected reference mathematical model, while $SS_{p}$ corresponds to the more complex model. The number of degrees of freedom represents the difference between the amount of experimental data, *n*, and the number of parameters, $df_{p}=n-p$ and $df_{q}=n-q$.

The analysis makes sense when the two models are nested, i.e., the model with a lower number of parameters can be considered as degenerated from the model with more parameters, by keeping the number of parameters constant.

In the case of closely related fitting performances, the decisive criterion is connected with the involved phenomena. It is preferable to use the model whose initial and boundary conditions are compatible with the structure and properties of the concerned supramolecular system. However, if these correlations are difficult to make, the correlation coefficient, information criteria, and Fisher test together have to be applied for selection of the most performant model. 

Last but not least, it has to be considered if fitting works for the partial or full-range time of the experiments. 

Many papers concluded with the application of Higuchi’s law, without looking at conditions used to derive the law were fulfilled by the respective experimental conditions in classical formulations [211,212,213]. 

## 5. Conclusions

The predictability of release kinetics of active substances represents an essential characteristic applied to supramolecular carrier systems in order to be accepted as drugs. Both safety and efficacy depend on the rate and extent of availability of active substances at the place of absorption and at the site of action. 

The measuring, the modeling, and the prediction of release kinetics represent research of high complexity, implying an in-depth understanding of physicochemical, physiological, and mathematical aspects. Unavoidably, almost all approaches start from one domain, and from one scientific language, while neglecting the other domains. Although many papers, many books, and many reviews were written, all of these satisfied only specific cases from one or two marginal sub-domains. Consequently, all future papers and reviews are welcome, but they surely cannot overcome some of these irreducible difficulties. 

On the other hand, it is continually emphasized that the more complex the model is, the more data are needed in order to validate it. Uncertainty, lack of uniqueness, and robustness increase with the number of parameters.

Furthermore, since the complexity and diversity of mechanistic models is huge, a clustering of these models as a function of boundary conditions, as tried in this paper, would probably allow a better understanding of the phenomena, and more efficient research for new models. 

## Figures and Tables

**Table 1:** Examples of the application of empirical models in describing release kinetics from micro-sized polymeric carriers.

Drug	Supramolecular System	Main Excipients	Release Experiment	Empirical Model	Reference
Cefpodoxime proxetil	Micro-balloons (hollow microspheres)	Hydroxypropylmethyl cellulose (HPMC) ethyl cellulose (EC)	Method (M): United States Pharmacopoeia (USP) paddle apparatus Dissolution medium (DM): 0.1 N HCl (pH 1.2)	Higher values of correlation coefficients were obtained in the case of Higuchi’s square root of time kinetic treatment; diffusion was the predominant mechanism of drug release.	[156]
Nimodipine Coumarin	Microparticles	PLGA	DM: 50/50 (*w*/*w*) mixture of phosphate-buffered saline (PBS), pH 7.4 and ethanol	Higuchi model	[157]
Ethinyl estradiol (EE) Drospirenone (DRSP)	Microparticles	PLGA	M: dialysis sac method DM: USP phosphate buffer pH 7.4 + 8% 2-Hydroxypropyl--β-cyclodextrin	EE release from PLGA microparticles was faster than DRSP release; EE release is assumed to be primarily controlled by drug diffusion.	[158]
Sodium fluorescein (hydrophilic compound)	Spray-dried microparticle	Poly(glycerol adipate-*co*-ω-pentadecalactone), l-arginine, l-leucine	DM: PBS, pH 7.4 (*n* = 3)	Higuchi model	[159]
Levonorgestrel	Microparticles	PLGA; Methocel Polyvinyl alcohol	DM: 0.9% sodium chloride + 0.5% sodium dodecyl sulfate	Release kinetics followed predominantly a zero-order release profile.	[160]
Anastrozole	Microparticles	PLGA	M: modified dialysis method DM: 0.1 N HCl (pH 1.2) and phosphate buffer (pH 7.4).	An initial burst release phase was followed by a gradual release phase with good correlation coefficients for the Higuchi model.	[161]
Centchroman	Microparticles	Glutaraldehyde Glyoxal	NA	A burst release of 29% centchroman within an initial period of 40 h was seen, and the remaining 70% was released in the next 60 h following zero-order release kinetics.	[162]
5-fluorouracil (5-FU)	Microspheres	Bovine serum albumin Galactosylated chitosan (coating)	M: dynamic dialysis DM: phosphate buffered saline (pH 7.4, PBS)	Attenuated burst release in comparison with uncoated microspheres. Release followed Higuchi’s square root model.	[163]
Methotrexate (MTX) 5-fluorouracil (5-FU)	Microspheres	Chitosan	DM: PBS, pH 7.4	Biphasic release (more prominent for MTX microspheres). 5-FU release followed Higuchi’s model, whereas MTX was released more slowly with a combination of first-order kinetics and Higuchi’s square-root model	[164]
Vitamin B_12_	Microparticles	Bovine serum albumin (BSA)	M: dialysis technique DM: pH 2, pH 6 and pH 10 buffers	First stage: power law and Weibull equations. The second stage: super case II transport mechanism, as a result of diffusion, relaxation, and erosion. Application of Hixson–Crowell model confirmed the erosion mechanism.	[165]
Aspirin	Microcapsules	Ethyl cellulose, Cellulose Acetate Phthalate	M: USP apparatus 2 DM: pH-1.2 for 2 h followed by acetate buffer at pH 6.0 for 7 h	The best fit was the Higuchi model, indicating diffusion-controlled release. The *n* in Korsemeyer–Peppas model varied between 0.5 and 0.7, suggesting a diffusion-controlled release.	[166]

**Table 2:** Examples of application of empirical models in describing release kinetics from nano-sized polymeric carriers.

Drug	Supramolecular System	Main Excipients	Release Experiment	Empirical Model	Reference
Docetaxel	Nanoparticles	Chitosan	Method (M): dialysis sac method Dissolution medium (DM): PBS pH 7.4	Higuchi’s square-root and Korsmeyer–Peppas; 0.45 ≤ *n* ≤ 0.89 indicates a combination of both diffusion of drug through the polymer and dissolution of the polymer.	[167]
Ofloxacin	Nanoparticles	Carboxymethyl gum kondagogu; Chitosan	M: dialysis sac method DM: phosphate buffer solution pH 7.4	Higuchi model; ‘*n*’ exponent of Peppas equation (*n* < 0.43) suggested diffusion-controlled mechanism.	[168]
Aceclofenac	Nanoparticles	Eudragit RL 100-	M: dialysis sac method DM: Sorenson’s phosphate buffer	Higuchi model (0.43 < *n* < 0.85)	[169]
Ellagic Acid	Biodegradable nanoparticles	PLGA polycaprolactone (PCL)	M: dialysis technique DM: phosphate buffer pH 7.4	An initial burst release was followed by Higuchi’s square-root pattern in the case of PLGA and PCL nanoparticles.	[170]
Estradiol	Nanoparticles	PLGA	M: dialysis technique DM: phosphate buffer pH 7.4	Zero order for low-molecular-weight nanoparticles; it was considered that degradation plays a dominant role and controls the release rate. High-molecular-weight nanoparticles showed the best fit into the Higuchi’s model.	[171]
Doxorubicin	Nanoparticles	Gelatin cross-linked with genipin Fe_3_O_4_	DM: PBS pH 7.4	A correlation between the quantity of released drug and swelling of the nanoparticles was established using a power-law model.	[172]
Chloroquine phosphate	Nanoparticles	Gelatin	DM: PBS pH 7.4 and distilled water	Fick’s power law allowed establishing a correlation between the quantity of released drug and swelling of the nanoparticles.	[173]
Indomethacin	Nanocapsules	Pluronic F127 Polylactide (PLA) Labrafac CC	M: dialysis technique DM: PBS pH 7.4	The release pattern was found to follow a power-law model, with *n* values ranging between 0.35 and 1.03 (depending on the preparation method).	[174]
Tigecycline	Nanoparticles	Calcium phosphate (CP) PLGA	DM: physiological solution at 37 °C under static conditions	The tigecycline content was released within a 35-day period. The in vitro data were best fitted with the Weibull model, and the release was defined as non-Fickian transport.	[141]
Moxifloxacin	Nanosuspensions	PLGA	M: USP apparatus 1 DM: simulated tear fluid (pH 7.4)	All formulations followed Korsemeyer–Peppas release kinetics with *n* values between 0.45 and 0.89 (anomalous behavior).	[175]

**Table 3:** Examples of experiments concerning release from liquid crystals, described by empirical models.

Drug	Supramolecular System	Main Excipients	Release Experiment	Empirical Model	Reference
Alpha lipoic acid (ALA)	Cubosomes loaded gel	Glycerol monooleate (GMO) Poloxamer P407	M: USP Apparatus 5, paddle over disk assembly DM: hydro-alcoholic solution (1:1), 700 mL	Higuchi model ALA release from cubosomes in gels was shown to be primarily controlled by diffusion through the matrix.	[192]
Doxorubicin	Bicontinuous lipidic cubic phases (LCPs)	GMO Phytantriol (PT)	DM: pH 7.4 and pH 5.8 buffer	Higuchi model was *n* > 0.5 in all cases, indicating non-Fickian anomalous transport in which both diffusion and matrix effects.	[193]
Capsaicin	Cubic phase gels	GMO: propylene glycol (1,2-propanediol, PG): water	DM: isotonic phosphate buffered solution (PBS)	Release kinetics were determined to fit Higuchi’s square-root equation indicating that the release was under diffusion control. The calculated diffusion exponent showed the release from cubic phase gels was anomalous transport (*n* = 0.57–0.60)	[194]
Salicylic acid	Cubic phase gels	GMO Myverol 18–99^®^ distilled monoglycerides	M: USP app I DM: Isotonic phosphate buffer	Release mechanism could be fitted to both Higuchi and first-order models.	[195]
2-pyrrolidone (model)	In situ cubic phase forming monoglyceride drug delivery systems	Monoglyceride (GMO or glycerol monolinoleate) Cosolvents (ethanol, PEG 300, 2-pyrrolidone, DMSO)	DM: 0.1 M phosphate buffer, pH 7.4, with 0.1% sodium azide as preservative	The release of oligonucleotide from the fully swollen cubic phase matrix followed a diffusion-controlled release mechanism square-root Higuchi model in 24-h intervals for all formulations.	[196]
Carbamazepine	Nanoemulsion	Castor oil; Lipophilic emulsifier (lecithin or polyoxyl 35 castor oil); Tween 80	M: dialysis technique DM: phosphate buffer pH 7.4	Higuchi model best characterized the release profiles for the nanoemulsions and for the free drug, and drug release was described as a diffusion process based on Fick’s law.	[197]
l-glutathione	Microemulsions Liquid crystal systems	-	NA	Higuchi model	[198]

**Table 4:** Examples of experiments concerning release from solid lipid nanoparticles and lipid dosage forms, described by empirical models.

Drug	Supramolecular System	Main Excipients	Release Experiment	Empirical Model	Reference
Etofenamate	Solid Lipid Nanoparticles (SLN)	Compritol 888 ATO Precirol ATO 5	NA	Higuchi model for Compritol 888 ATO SLNs; Zero-order release for Precirol ATO 5 SLNs	[202]
Curcuminoids	SLN	Poloxamer 188 Dioctyl sodium sulfosuccinate Stearic acid Glyceryl monostearate	M; vertical Franz diffusion cells DM: 50% (*v*/*v*) ethanol	25% burst release of the curcuminoids within 10 min followed by controlled release pattern following Higuchi’s square-root model for 12 h	[203]
Bixin	SLN	Trimyristin Glycerol monostearate	M: diffusion using Franz diffusion cells Receptor medium: Sorensen buffer pH 7.7	The release was first-order diffusion-controlled. The *n*-values obtained from the Korsmeyer–Peppas model (*n* = 0.697) indicated the release mechanism was non-Fickian type.	[204]
Gatifloxacin	SLN	Stearic acid (SA)/ Compritol/Gelucire Poloxamer-188 Sodium taurocholate	M: Automated transdermal diffusion cells Receptor medium: phosphate buffer (pH 7.4)	The release pattern was found to follow Korsmeyer–Peppas model (*n* = 0.15).	[205]

## Appendix A

### Diffusion in a Domain Bordered by Two Interfaces where Concentration is Kept Constant

Here, we consider the release from (or into) a domain of thickness 2ℓ, starting from an initial concentration $c_{1}$ into an environment where the concentration maintains constant over time $c_{0}$.

If concentration at the point *x* in the matrix at the moment *t* is *c*(*x*,*t*), the initial and boundary conditions can be written in the form

(A1) $$\left|\begin{array}{c} c_{0}\\ c_{1}\\ c_{0} \end{array}\right|\;\begin{array}{cc} x=2\ell , & c(2\ell ,t)=c_{0}\\ t=0 & c(x,0)=c_{1}\\ x=0 & c(0,t_{0})=c_{0} \end{array}.$$

The diffusion equation to be solved is the second law of Fick.

(A2) $$\frac{\partial C}{\partial t}=D\frac{\partial^{2}C}{\partial x^{2}}.$$

By making the change of variable,

(A3) $$\theta =\frac{c-c_{0}}{c_{0}-c_{1}},$$

and applying the general rules of differential calculus, it can be easily obtained that $\frac{\partial \theta }{\partial t}=\frac{1}{c_{1}-c_{0}}-\frac{\partial c}{\partial t}$ and $\frac{\partial^{2}\theta }{\partial x^{2}}=\frac{1}{c_{1}-c_{0}}-\frac{\partial^{2}c}{\partial x^{2}}$, and substituting into the equation of *C*(*x*,*t*) results that θ satisfies the same equation as *C*(*x*,*t*).

(A4) $$\frac{\partial \theta }{\partial t}=D\frac{\partial^{2}\theta }{\partial x^{2}}.$$

With initial and boundary conditions modified,

(A5) $$\;\begin{array}{cc} x=2\ell , & \theta (2\ell ,t)=0\\ t=0 & \theta (x,0)=1\\ x=0 & \theta (0,t_{0})=0 \end{array}.$$

We are looking for a solution in the form of a product between a function only of *x* and a function only of *t*. In these conditions, the method is sometimes called “the method of separation of variables”.

(A6) θ(x,t)=X(x)·T(t).

Substituting in the diffusion equation, we obtain

(A7) T’X=DTX’’,

which can be rewritten in the form

(A8) $$\frac{T’}{DT}=\frac{X’’}{X’}=-\lambda ,$$

where −λ is the common value of the two ratios. The system is equivalent to two differential equations. The first of these $\frac{T’}{T}=-\lambda D$ is with separate variables and integrates immediately.

(A9) $$\int \frac{T’}{T}dt=-\int \lambda Ddt.$$

lnT=−λDt+lna, where lna is the constant of integration. Consequently, *T*(*t*) can be expressed in the form

(A10) $$T(t)=a\cdot e^{-\lambda D\;t}.$$

General solutions of the second equation X"+λX=0 have different forms depending on the sign of λ.

(a) λ<0,

(A11) $$X(x)=k_{1}e^{\sqrt{-\lambda }x}+k_{2}e^{-\sqrt{-\lambda }x}.$$

By imposing that the solution satisfies the boundary conditions, we get

$$x=0\Rightarrow \theta (0,t)=0\Rightarrow X(0)T(t)=0\;i.e.\;\left(k_{1}e^{\sqrt{-\lambda }0}+k_{2}e^{-\sqrt{-\lambda }0}\right)ae^{-\lambda Dt}=0.$$

Furthermore, because $ae^{-\lambda Dt}\neq 0$ and $e^{0}=1$, we have $k_{1}+k_{2}=0$ and $k_{2}=-k_{1}$.

At the other face of the tablets, the above equation becomes

(A12) $$x=2\ell \Rightarrow \theta (2\ell ,t)=0\Leftrightarrow k_{1}\left(e^{2\sqrt{-\lambda }0}-e^{-2\sqrt{-\lambda }0}\right)e^{-\lambda Dt}=0.$$

Since no parenthesis nor exponential of *t* can be zero, it remains to take $k_{1}=0$. Hence, it was obtained that X(x)=0, i.e., a trivial solution.

(b) λ>0. In this case the general solution can be written in the form

(A13) $$X(x)=k_{1}\cos\sqrt{\lambda }x+k_{2}\sin\sqrt{\lambda }x.$$

The solution has to satisfy the initial and boundary conditions as follows:

x=0⇒θ(0,t)=0⇒X(0)T(t)=0,

Thus,

$$X(0)T(t)=\left(k_{1}\cos\sqrt{\lambda }\cdot 0+k_{2}\sin\sqrt{\lambda }\cdot 0\right)e^{-\lambda Dt}=k_{1}e^{-\lambda Dt}=0.$$

Since the exponential cannot be null, then $k_{1}=0$.

$$\begin{array}{l} x=2\ell \Rightarrow \theta (2\ell ,t)=0\Rightarrow \left(k_{2}\sin\sqrt{\lambda }\cdot 2\ell \right)e^{-\lambda Dt}=0\Rightarrow \\ \Rightarrow \sin2\ell \sqrt{\lambda }=0\Rightarrow 2\ell \sqrt{\lambda }=n\pi , \end{array}$$

which means practically a condition imposed to λ.

(A14) $$\lambda_{n}=\frac{n^{2}\pi^{2}}{4\ell^{2}}.$$

Thus, for every *n*, we obtained a function which meets the initial and boundary conditions and is a solution of the diffusion equation. We can further write the general solution as a linear combination of these particular solutions.

(A15) $$X(x)=\sum_{n\geq 0}c_{n}\sin\frac{n\pi }{2\ell }x.$$

In order to obtain the values of the constants, we impose a general solution that satisfies the initial condition θ(x,0)=1.

$$\left(\sum_{n\geq 0}c_{n}\sin\frac{n\pi x}{2\ell }\right)e^{-\lambda D0}=1\Rightarrow \left(\sum_{n\geq 0}c_{n}\sin\frac{n\pi x}{2\ell }\right)=1.$$

By multiplying both members through $\sin\frac{m\pi x}{2\ell }$ and integrating between 0 and 2ℓ, we get

(A16) $$\frac{1}{\ell }\cdot \int_{0}^{2\ell }\left(\sum c_{n}\sin\frac{n\pi x}{2\ell }\right)\sin\frac{m\pi x}{2\ell }dx=\frac{1}{\ell }\cdot \int_{0}^{2\ell }1\cdot \sin\frac{m\pi x}{2\ell }dx.$$

As can be easily verified, $\frac{1}{\ell }\cdot \int_{0}^{2\ell }\sin\frac{n\pi x}{2\ell }\sin\frac{m\pi x}{2\ell }dx=0$; thus, except for *n = m*, the entire sum will reduce only to the term “m”.

(A17) $$\begin{array}{l} c_{m}=\frac{1}{\ell }\int_{0}^{2\ell }\sin\frac{m\pi x}{2\ell }\cdot 1\;dx=\frac{1}{\ell }\left(-\frac{2\ell }{m\pi }\right){\cos\frac{m\pi x}{2\ell }|}_{0}^{2\ell }=\\ =\frac{1}{\ell }\left(-\frac{2\ell }{m\pi }\right)\left(\cos m\pi -1\right)=-\frac{2}{m\pi }\left[{\left(-1\right)}^{m}-1\right] \end{array}$$

The term in parenthesis is null for even numbers of *m* and *m* = −2 for odd numbers of *m*. We can then obtain the solution of the Cauchy problem.

(A18) $$\frac{c-c_{0}}{c_{1}-c_{0}}=\frac{4}{\pi }\sum \frac{1}{2k+1}\sin\frac{\left(2k+1\right)\pi x}{2\ell }e{-}^{\frac{{\left(2k+1\right)}^{2}\pi^{2}t}{4\ell^{2}}}.$$
